# Survival after traumatic out-of-hospital cardiac arrest in Vietnam: a multicenter prospective cohort study

**DOI:** 10.1186/s12873-021-00542-z

**Published:** 2021-11-23

**Authors:** Son Ngoc Do, Chinh Quoc Luong, Dung Thi Pham, My Ha Nguyen, Tra Thanh Ton, Quoc Trong Ai Hoang, Dat Tuan Nguyen, Thao Thi Ngoc Pham, Hanh Trong Hoang, Dai Quoc Khuong, Quan Huu Nguyen, Tuan Anh Nguyen, Tung Thanh Tran, Long Duc Vu, Chi Van Nguyen, Bryan Francis McNally, Marcus Eng Hock Ong, Anh Dat Nguyen

**Affiliations:** 1 Center for Emergency Medicine, Bach Mai Hospital, 78 Giai Phong road, Phuong Mai ward, Dong Da district, Hanoi, 100000 Vietnam; 2 Department of Emergency and Critical Care Medicine, Hanoi Medical University, Hanoi, Vietnam; 3 Faculty of Medicine, University of Medicine and Pharmacy, Vietnam National University, Hanoi, Vietnam; 4 Department of Nutrition and Food Safety, Faculty of Public Health, Thai Binh University of Medicine and Pharmacy, Thai Binh, Vietnam; 5 Department of Health Organization and Management, Faculty of Public Health, Thai Binh University of Medicine and Pharmacy, Thai Binh, Vietnam; 6 Emergency Department, Cho Ray Hospital, Ho Chi Minh City, Vietnam; 7 Emergency Department, Hue Central General Hospital, Hue City, Thua Thien Hue Vietnam; 8 Intensive Care Department, Cho Ray Hospital, Ho Chi Minh City, Vietnam; 9 Department of Critical Care, Emergency Medicine and Clinical Toxicology, Faculty of Medicine, Ho Chi Minh City University of Medicine and Pharmacy, Ho Chi Minh City, Vietnam; 10 Intensive Care Department, Hue Central General Hospital, Hue City, Thua Thien Hue Vietnam; 11 Department of Emergency and Critical Care Medicine, Faculty of Medicine, University of Medicine and Pharmacy, Hue City, Thua Thien Hue Vietnam; 12 Department of Emergency Medicine, Emory University School of Medicine, Atlanta, GA USA; 13 Emory University Rollins School of Public Health, Atlanta, GA USA; 14 Department of Emergency Medicine, Singapore General Hospital, Singapore, Singapore; 15 Duke-NUS Medical School, Singapore, Singapore

**Keywords:** Emergency medical services, First-aid, Organized trauma system of care, Out-of-hospital cardiac arrest, PAROS study, Pre-hospital care, Road traffic injuries, Trauma center, Trauma, Traumatic OHCA

## Abstract

**Background:**

Pre-hospital services are not well developed in Vietnam, especially the lack of a trauma system of care. Thus, the prognosis of traumatic out-of-hospital cardiac arrest (OHCA) might differ from that of other countries. Although the outcome in cardiac arrest following trauma is dismal, pre-hospital resuscitation efforts are not futile and seem worthwhile. Understanding the country-specific causes, risk, and prognosis of traumatic OHCA is important to reduce mortality in Vietnam. Therefore, this study aimed to investigate the survival rate from traumatic OHCA and to measure the critical components of the chain of survival following a traumatic OHCA in the country.

**Methods:**

We performed a multicenter prospective observational study of patients (> 16 years) presenting with traumatic OHCA to three central hospitals throughout Vietnam from February 2014 to December 2018. We collected data on characteristics, management, and outcomes of patients, and compared these data between patients who died before hospital discharge and patients who survived to discharge from the hospital.

**Results:**

Of 111 eligible patients with traumatic OHCA, 92 (82.9%) were male and the mean age was 39.27 years (standard deviation: 16.38). Only 5.4% (6/111) survived to discharge from the hospital. Most cardiac arrests (62.2%; 69/111) occurred on the street or highway, 31.2% (29/93) were witnessed by bystanders, and 33.7% (32/95) were given cardiopulmonary resuscitation (CPR) by a bystander. Only 29 of 111 patients (26.1%) were taken by the emergency medical services (EMS), 27 of 30 patients (90%) received pre-hospital advanced airway management, and 29 of 53 patients (54.7%) were given resuscitation attempts by EMS or private ambulance. No significant difference between patients who died before hospital discharge and patients who survived to discharge from the hospital was found for bystander CPR (33.7%, 30/89 and 33.3%, 2/6, *P* > 0.999; respectively) and resuscitation attempts (56.3%, 27/48, and 40.0%, 2/5, *P* = 0.649; respectively).

**Conclusion:**

In this study, patients with traumatic OHCA presented to the ED with a low rate of EMS utilization and low survival rates. The poor outcomes emphasize the need for increasing bystander first-aid, developing an organized trauma system of care, and developing a standard emergency first-aid program for both healthcare personnel and the community.

**Supplementary Information:**

The online version contains supplementary material available at 10.1186/s12873-021-00542-z.

## Introduction

Traumatic out-of-hospital cardiac arrest (OHCA) is one of the leading causes of death, especially in young people throughout the world [1, 2]. Traumatic OHCA is defined as the loss of functional cardiac mechanical activity in association with an absence of systemic circulation, caused by an injury (e.g., blunt or penetrating trauma, burns, etc.), and occurring outside of a hospital setting [3, 4]. In the high-income countries (HICs), more than 5 million traumas occur each year and about 7% of them are complicated by OHCA [1, 5]. In the Asia-Pacific countries, traumatic OHCA accounted for 3.2% (13/450) - 22.2% (77/573) of people with OHCA [4].

The outcome in cardiac arrest following trauma is dismal and, on this basis, the American College of Surgeons Committee on Trauma (ACS COT) guidelines state that cardiopulmonary resuscitation (CPR) of a traumatic OHCA should be considered futile if the patient has unorganized electric activity without a pulse [6, 7]. However, previous studies seem to show that when CPR was started in combination with aggressive, advanced cardiac life support (ACLS), performed either by paramedics or other medical teams, patients have a more favorable outcome and may survive on discharge from the hospital [5, 8–13]. In Asia-Pacific countries, emergency medical services (EMS) systems are underdeveloped and vary considerably [14]. Survival outcomes for OHCA in Asia differ considerably and these variations may be related to differences in the patients and the EMS system [4]. These differences suggest that survival outcomes in many countries can also be improved with interventions to enhance EMS, such as increasing bystander first-aid in trauma through community-based first-aid training programs, [15, 16] building a trauma system, [17] and improving post-resuscitation care.

Vietnam is a low- and middle-income country (LMIC), ranked 15th in the world and 3rd in Southeast Asia by population with 96.462 million people [18]; the majority of injury-related deaths are associated with traffic crashes [19, 20]. Road traffic injuries (RTIs) are becoming a major public health issue [19–23]. These injuries occur more frequently due to rapid economic growth and motorization in the past 25 years; indeed, the number of road traffic fatalities nationally rose from 4907 in 1994 to 7624 in 2019 [21–23]. In contrast, annual injury-related deaths are often associated with unintentional injuries and the number of injury-related deaths fell from 1035/18,481 (5.6%) in 2012 to 858/21,446 (4%) in 2019 in Singapore [24]. The Government of Vietnam introduced a nationwide policy on the EMS system in 2008, however, pre-hospital services are not well developed and only a few places, such as urban areas, have a functioning EMS system. Additionally, the lack of a trauma system of care prevents integration of pre-hospital and hospital treatment protocols and the collection of clinical data for surveillance, quality improvement and research related activities [21, 25–28]. Moreover, the ambulances, trained and accredited medical staff, and life-saving equipment available are limited, and medical oversight and regular monitoring of quality indicators are uncommon [27, 29]. As a result, the staff of the EMS is often overworked and not able to respond promptly to emergencies [27–31]. Furthermore, although national health insurance was established in 1992 to improve access to health care and mitigate the negative impact of user fees introduced in 1989, neither EMS nor private ambulance services are currently covered by health insurance.

Data are lacking on evidence-based performance measures for EMS in Vietnam, particularly emergency response time [27]. A previous study has shown that only 4% (3/75) of patients with injury were transported to the hospital by ambulances [25]. Most patients are brought by taxi, private vehicle, or motorbike, usually with no first-aid having been provided [21, 25, 26, 30, 31]. Thus, basic (BLS) and advanced life support (ALS) treatment is often delayed for patients with life-threatening illnesses or injuries until they arrive at the hospital.

Understanding the country-specific causes, risk, and prognosis of traumatic OHCA is important to reduce mortality in Vietnam. The aim of this study, therefore, was to investigate the survival rate from traumatic OHCA and to measure the critical components of the chain of survival following a traumatic OHCA in the country.

## Methods

### Study design and setting

This multicenter prospective observational study is part of the Pan-Asian Resuscitation Outcomes Study (PAROS), Clinical Research Network, which collects data on patients with OHCA admitted to hospital emergency departments (EDs) in countries of Asia [31–33]. In this study, we used only data for Vietnam. The hospitals in Vietnam participating in the PAROS study are three public sector tertiary hospitals in the three largest cities of the country: Hanoi (northern Vietnam) which serves an estimated 10 million people; Hue (central Vietnam) which serves 1.154 million people; and Ho Chi Minh City (southern Vietnam) which serves 13 million people. The hospitals receive patients from all parts of each city.

Several ambulance services are available in Vietnam, but only one emergency service has an emergency number (i.e., telephone 115), trained and accredited medical staff, life-saving equipment, medical oversight and quality indicators that are regularly monitored [27, 29]. Several other private organizations provide so-called emergency transportation but with limited medical interventions at the scene or during transportation [34]. For this study, we categorized type of pre-hospital transportation into two groups: EMS, which refers to ambulances dispatched by an EMS dispatch center; and non-EMS, which refers to private ambulances, own or private transport, or public transport. We defined a private ambulance as an ambulance that was not dispatched by an EMS dispatch center. Own or private transport includes transport in vehicles of family members, relatives, neighbors or passers-by. Public transport includes taxis, buses or other types of public transport.

### Participants

This study included all patients (older than 16 years) presenting with OHCA to the EDs of the three hospitals. Patients with non-traumatic OHCA were excluded. We defined a case of OHCA as a person who was unresponsive, not breathing and without a pulse outside the hospital setting [35–37]. In addition, we also defined a traumatic OHCA as an injury (e.g., blunt, penetrating, or burn injury, etc.) outside the hospital setting which resulted in cardiac arrest [38]. A physician confirmed the diagnosis either in the ambulance or in the ED. We excluded patients for whom resuscitation was not attempted by staff of the EMS or private ambulance at the scene or on the way to hospital and who were immediately pronounced dead (because of decapitation, rigor mortis, lividity and do not resuscitate orders) at the ED. However, we included patients on whom resuscitation was attempted but who were later pronounced dead before they reached the hospital.

### Data collection and management

We used a standardized classification and case record form to collect data on common variables. The data dictionary of the PAROS study is available as an online supplement of a previously published paper [4]. We extracted data from emergency dispatch records, ambulance patient case notes, and ED and in-hospital records. Data were entered into the database of the PAROS study by the electronic data-capture system. Patient identifiers were not entered in the database to protect patient confidentiality. We then merged the data sets for the three hospitals. Each hospital contributed 5 years of data from February 2014 to December 2018.

### Variables

We included variables based on Utstein recommendations, [38, 39] such as information on: (i) bystander CPR; (ii) availability of public access defibrillator; (iii) response times; (iv) provision of ACLS (e.g., intravenous drugs, advanced airway management including endotracheal intubation, or alternative airway devices); and (v) specialized post-resuscitation care (e.g., hypothermia). We also collected data on the location of the OHCA (e.g., home, public area) and system variables which are available in Additional file 1.

### Outcomes

The primary outcome was survival to hospital discharge. We also examined the following secondary outcomes: return of spontaneous circulation (ROSC), survival to hospital admission and neurological status on discharge from hospital [40].

### Statistical analyses

We used IBM® SPSS® Statistics 25.0 (IBM Corp., Armonk, United States of America) for data analysis. We report data as number and percentages for categorical variables and medians and interquartile ranges (IQRs) or means and standard deviations (SDs) for continuous variables. Comparisons were made among type of pre-hospital care, and between death before hospital discharge and survival to discharge from the hospital for each variable, using the χ2 test or Fisher exact test for categorical variables and the Mann–Whitney U test, Kruskal-Wallis test, one-way analysis of variance for continuous variables. For all analyses, significance levels were two-tailed, and we considered *P* < 0.05 as statistically significant.

## Results

Data on 779 patients with OHCA were submitted to the database of the PAROS study during the study period. Of these patients, we excluded 25 patients aged 16 years or younger, and 639 with non-traumatic injury. We also excluded 1 patient (0.87%; 1/115) because of long pre-hospital time (i.e., longer than one day), which might imply simple input errors or specific pre-hospital circumstances. In addition, we excluded some patients because of missing or unknown data: one without date and time data of arrival at the ED (0.87%; 1/115) and two without pre-hospital information (1.74%; 2/115).

**Fig. 1:**
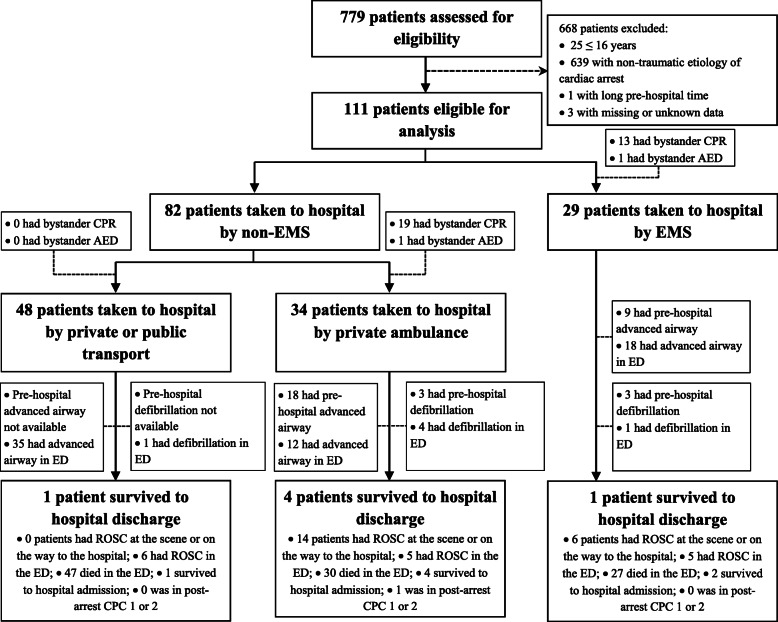
Flowchart of transportation to the hospital, treatment, and outcome of patients with traumatic out-of-hospital cardiac arrest included in the study, Vietnam, February 2014–December 2018 (**AED**, automated external defibrillation; **CPC**, cerebral performance category; **CPR**, cardiopulmonary resuscitation; **ECMO**, extracorporeal membrane oxygenation; **ED**, emergency department; **EMS**, emergency medical services; **ROSC**, return of spontaneous circulation)

Thus, we included 111 patients with traumatic OHCA in our analyses (Fig. 1 and Table 1). Of these patients, 92/111 (82.9%) were men and the mean age was 39.27 years (SD: 16.38). Most traumatic OHCAs occurred on the street or highway (69/111; 62.2%) followed by at home (20/111; 18.0%); 45.2% (42/93) of which were witnessed by EMS or private ambulance, and 31.2% (29/93) were witnessed by bystanders. Among 20 patients who were collected the time-stamped data on cardiac arrest events and initiation of CPR, the time from cardiac arrest to initiation of CPR was 2.68 (SD, 5.66) min (Table 1). Only 32/95 (33.7%) patients received bystander CPR and 2/53 (3.8%) received bystander automated external defibrillation (AED). In a total of 30 first documented arrest rhythms, there were 19 (63.3%) shockable rhythms and 11 (36.7%) non-shockable rhythms. In addition, only 27/30 patients received pre-hospital advanced airway management, 29/53 were given resuscitation attempts by EMS or private ambulance, 6/30 received pre-hospital defibrillation, and epinephrine was given to 24.3% (27/111) of patients before reaching the hospital. Upon ED admission, only 58.6% (65/111) of patients received advanced airway management and epinephrine was given to 91.0% (101/111) of patients with traumatic OHCA.

**Table 1:** Characteristics, management and outcomes of patients with traumatic out-of-hospital cardiac arrest according to the type of transportation to the hospital, Vietnam, February 2014–December 2018

Characteristics	All cases	Non-EMS		EMS	*P*-value^a^
		Private or public transport	Private ambulance		
**Hospital participated**	*n* = 111	*n* = 48	*n* = 34	*n* = 29	< 0.001
Bach Mai hospital, no. (%)	33 (29.7)	13 (27.1)	19 (55.9)	1 (3.4)	
Hue hospital, no. (%)	26 (23.4)	21 (43.8)	3 (8.8)	2 (6.9)	
Cho Ray hospital, no. (%)	52 (46.8)	14 (29.2)	12 (35.3)	26 (89.7)	
**Patient related**	*n* = 111	*n* = 48	*n* = 34	*n* = 29	
Age (year), mean (SD)	39.27 (16.38)	38.31 (14.74)	40.71 (17.59)	39.17 (17.91)	0.931
Gender, no. (%)	*n* = 111	*n* = 48	*n* = 34	*n* = 29	0.993
Male	92 (82.9)	40 (83.3)	28 (82.4)	24 (82.8)	
Female	19 (17.1)	8 (16.7)	6 (17.6)	5 (17.2)	
Past medical history, no. (%)	*n* = 67	*n* = 23	*n* = 27	*n* = 17	
Heart disease	2 (3.0)	1 (4.3)	1 (3.7)	0	> 0.999
Diabetes	2 (3.0)	0	2 (7.4)	0	0.335
Hypertension	6 (9.0)	2 (8.7)	4 (14.8)	0	0.268
Renal disease	2 (3.0)	1 (4.3)	0	1 (5.9)	0.512
Respiratory disease	1 (1.5)	1 (4.3)	0	0	0.597
Other	9 (13.4)	2 (8.7)	7 (25.9)	0	0.039
**Event related**					
Location type, no. (%)	*n* = 111	*n* = 48	*n* = 34	*n* = 29	0.001
Home residence	20 (18.0)	11 (22.9)	8 (23.5)	1 (3.4)	
Healthcare facility	4 (3.6)	0	4 (11.8)	0	
In EMS/Private ambulance	6 (5.4)	0	5 (14.7)	1 (3.4)	
Industrial place	8 (7.2)	2 (4.2)	2 (5.9)	4 (13.8)	
Street/Highway	69 (62.2)	32 (66.7)	14 (41.2)	23 (79.3)	
Transport center	4 (0.9)	0	1 (2.9)	0	
Other	3 (2.7)	3 (6.3)	0	0	
Time of the day, no. (%), n = 60	34 (56.7)	6 (54.5)	15 (65.2)	13 (50.0)	0.556
Arrest witnessed by, no. (%)	*n* = 93	*n* = 30	*n* = 34	*n* = 29	< 0.001
Not witnessed	22 (23.7)	14 (46.7)	7 (20.6)	1 (3.4)	
Bystander (Lay person)	10 (10.8)	8 (26.7)	1 (2.9)	1 (3.4)	
Bystander (Family)	5 (5.4)	0	4 (11.8)	1 (3.4)	
Bystander (Healthcare provider)	14 (15.0)	8 (26.7)	5 (14.7)	1 (3.4)	
EMS/Private ambulance	42 (45.2)	0	17 (50.0)	25 (86.2)	
First arrest rhythm, no. (%)	*n* = 30	–	*n* = 21	*n* = 9	0.687
Shockable rhythm^b^	19 (63.3)	not available	14 (66.7)	5 (55.6)	
Unshockable rhythm	11 (36.7)	not available	7 (33.3)	4 (44.4)	
Prehospital intervention, no. (%)					
Bystander CPR, *n* = 95	32 (33.7)	0	19 (55.9)	13 (44.8)	< 0.001
Prehospital defibrillation, *n* = 30	6 (20)	not available	3 (14.3)	3 (33.3)	0.329
Bystander AED applied, *n* = 111	2 (1.8)	0	1 (2.9)	1 (3.4)	0.320
ED defibrillation performed, no. (%), *n* = 111	6 (5.4)	1 (2.1)	4 (11.8)	1 (3.4)	0.200
**System related**					
Resuscitation attempted by EMS/private ambulance, no. (%), *n* = 53	29 (54.7)	not available	20 (58.8)	9 (47.4)	0.422
Time to CPR at scene (min), mean (SD), *n* = 20	2.68 (5.66)	not available	2.53 (4.74)	2.95 (7.52)	0.553
**Therapeutic related**					
Pharmacotherapy, no. (%)	*n* = 111	*n* = 48	*n* = 34	*n* = 29	
Epinephrine (at scene)	27 (24.3)	0	19 (55.9)	8 (27.6)	< 0.001
Epinephrine (at ED)	101 (91.0)	44 (91.7)	30 (88.2)	27 (93.1)	0.837
Prehospital advanced airway, no. (%), *n* = 30	27 (90.0)	not available	18 (85.7)	9 (100)	0.534
Advanced airway used at ED, no. (%), *n* = 111	65 (58.6)	35 (72.9)	12 (35.3)	18 (62.1)	0.003
Post-resuscitation care, no. (%)					
Hypothermia therapy initiated, *n* = 111	2 (1.8)	0	2 (5.9)	0	0.158
**Outcomes**					
ROSC, no. (%)	*n* = 111	*n* = 48	*n* = 34	*n* = 29	
ROSC at scene/en-route,	20 (18.0)	0	14 (41.2)	6 (20.7)	< 0.001
ROSC at ED,	16 (14.4)	6 (12.5)	5 (14.7)	5 (17.2)	0.889
Outcome of patient at ED, no. (%)	*n* = 111	*n* = 48	*n* = 34	*n* = 29	0.197
Died in ED	104 (93.7)	47 (97.9)	30 (88.2)	27 (93.1)	
Admitted	7 (6.3)	1 (2.1)	4 (11.8)	2 (6.9)	
Patient status, no. (%)	*n* = 7	*n* = 1	*n* = 4	*n* = 2	0.429
Died in the hospital	1 (14.3)	0	0	1 (50.0)	
Remains in hospital at 30th day post arrest	0	0	0	0	
Discharged alive	6 (85.7)	1 (100)	4 (100)	1 (50.0)	
Post arrest CPC 1 and 2, no. (%), *n* = 111	1 (0.9)	0	1 (2.9)	0	0.568

^a^Shows a comparison between “EMS”, “Private ambulance” and “Private or public transport”

^b^Shockable rhythm includes ventricular fibrillation, ventricular tachycardia, or unknown shockable rhythm

Abbreviations: *AED* automatic external defibrillation, *CPC* cerebral performance category; *CPR* cardiopulmonary resuscitation, *ED* emergency department, *EMS* emergency medical services, *ET* endotracheal tube, *LMA* laryngeal mask airway, *OHCA* out-of-hospital cardiac arrest, *ROSC* return of spontaneous circulation, *SD* standard deviation, *Time of the day* cardiac arrest occurred between 08:00 and 20:00

Of the 111 patients with traumatic OHCA, over two-fifths of the patients with traumatic OHCA (43.3%; 48/111) were taken to hospital by private or public transport, 30.6% (34/111) were taken by private ambulance, and only 26.1% (29/111) were taken by EMS (Table 1). Of these patients, 20 (18%) achieved ROSC at the scene of the cardiac arrest or on the way to the hospital and for 16 (14.4%) patients, spontaneous circulation returned in the ED (Table 1). Overall, 6.3% (7/111) of patients survived on hospital admission, and 5.4% (6/111) survived to discharge from the hospital; 0.9% (1/111) survived with good neurological function (cerebral performance category score 1 and 2) (Table 1).

There were statistically significant associations between types of transportation to hospital and: place where the cardiac arrest occurred; whether the cardiac arrest was witnessed or not; administration of pre-hospital interventions; and ROSC at the scene or on the way to the hospital (Table 1; *P* < 0.001). In contrast, there were no statistically significant differences between patients who died before hospital discharge (105 patients) and patients who survived on discharge from the hospital (6 patients) for the general characteristics, pre-hospital and in-hospital management, and outcome (Table 2).

**Table 2:** Characteristics, management and outcomes of patients with traumatic out-of-hospital cardiac arrest according to survival to hospital discharge, Vietnam, February 2014–December 2018

Characteristics	All cases	Died	Survived	*P*-value^a^
**Hospital participated**	*n* = 111	*n* = 105	*n* = 6	0.558
Bach Mai hospital, no. (%)	33 (29.7)	30 (28.6)	3 (50.0)	
Hue hospital, no. (%)	26 (23.4)	25 (23.8)	1 (16.7)	
Cho Ray hospital, no. (%)	52 (46.8)	50 (47.6)	2 (33.3)	
**Patient related**				
Age (year), mean (SD), *n* = 111	39.27 (16.38)	39.0 (16.41)	44.00 (16.51)	0.379
Gender, no. (%)	*n* = 111	*n* = 105	*n* = 6	> 0.999
Male	92 (82.9)	58 (82.9)	5 (83.3)	
Female	19 (17.1)	18 (17.1)	1 (16.7)	
Past medical history, no. (%)	*n* = 67	*n* = 61	*n* = 6	
Heart disease	2 (3.0)	2 (3.3)	0	> 0.999
Diabetes	2 (3.0)	2 (3.3)	0	> 0.999
Hypertension	6 (9.0)	4 (6.6)	2 (33.3)	0.086
Renal disease	2 (3.0)	2 (3.3)	0	> 0.999
Respiratory disease	1 (1.5)	1 (1.6)	0	> 0.999
Other	9 (13.4)	7 (11.5)	2 (33.3)	0.181
**Event related**				
Location type, no. (%)	*n* = 111	*n* = 105	*n* = 6	0.171
Home residence	20 (18.0)	17 (16.2)	3 (50.0)	
Healthcare facility	4 (3.6)	3 (2.9)	1 (16.7)	
In EMS/Private ambulance	6 (5.4)	6 (5.7)	0	
Industrial place	8 (7.2)	8 (7.6)	0	
Street/Highway	69 (62.2)	67 (63.8)	2 (33.3)	
Transport center	1 (0.9)	1 (1.0)	0	
Other	3 (2.7)	3 (2.9)	0	
Time of the day, no. (%), *n* = 60	34 (56.7)	31 (54.4)	3 (100)	0.251
Arrest witnessed by, no. (%)	n = 93	*n* = 87	n = 6	0.305
Not witnessed	22 (23.7)	21 (24.1)	1 (16.7)	
Bystander (Lay person)	10 (10.8)	10 (11.5)	0	
Bystander (Family)	5 (5.4)	4 (4.6)	1 (16.7)	
Bystander (Healthcare provider)	14 (15.1)	12 (13.8)	2 (33.3)	
EMS/Private ambulance	42 (45.2)	40 (46.0)	2 (33.3)	
First arrest rhythm, no. (%)	*n* = 30	*n* = 28	*n* = 2	0.520
Shockable rhythm^b^	19 (63.3)	17 (60.7)	2 (100)	
Unshockable rhythm	11 (36.7)	11 (39.3)	0	
Prehospital intervention, no. (%)				
Bystander CPR, *n* = 95	32 (33.7)	30 (33.7)	2 (33.3)	> 0.999
Prehospital defibrillation, *n* = 30	6 (20.0)	5 (17.9)	1 (50.0)	0.366
Bystander AED applied, *n* = 111	2 (1.8)	2 (1.9)	0	> 0.999
ED defibrillation performed, no. (%), *n* = 111	6 (5.4)	6 (5.7)	0	> 0.999
**System related**				
Types of prehospital transportation, no. (%)	*n* = 111	*n* = 105	*n* = 6	0.200
Private or public transport	48 (43.2)	47 (44.8)	1 (16.7)	
Private ambulance	34 (30.6)	30 (28.6)	4 (66.7)	
EMS	29 (26.1)	28 (26.7)	1 (16.7)	
Resuscitation attempted by EMS/private ambulance, no. (%), *n* = 53	29 (54.7)	27 (56.3)	2 (40.0)	0.649
Time to CPR at scene (min), mean (SD), *n* = 20	2.68 (5.66)	2.03 (4.99)	15	0.115
**Therapeutic related**				
Pharmacotherapy, no. (%)	*n* = 111	*n* = 105	*n* = 6	
Epinephrine (at scene)	27 (24.3)	25 (23.8)	2 (33.3)	0.632
Epinephrine (at ED)	101 (91.0)	96 (91.4)	5 (83.3)	0.440
Prehospital advanced airway, no. (%), *n* = 30	27 (90.0)	25 (89.3)	2 (100)	> >0.999
Prehospital advanced airway techniques, no. (%)	*n* = 27	*n* = 25	*n* = 2	> >0.999
Oral/Nasal ET	26 (96.3 )	24 (96.0)	2 (100)	
LMA	1 (3.7)	1 (4.0)	0	
Advanced airway used at ED, no. (%), *n* = 111	65 (58.6)	62 (59.0)	3 (50.0)	0.691
Post-resuscitation care, no. (%)	*n* = 111	*n* = 105	*n* = 6	
Hypothermia therapy initiated	2 (1.8)	0	2 (33.3)	0.002
**Outcomes**				
Outcome of patient at ED, no. (%)	*n* = 111	*n* = 105	*n* = 6	< 0.001
Died in ED	104 (93.7)	104 (99.0)	0	
Admitted	7 (6.3)	1 (1.0)	6 (100)	
Patient status, no. (%)	*n* = 7	*n* = 1	*n* = 6	0.143
Died in the hospital	1 (14.3)	1 (100)	0	
Remains in hospital at 30th day post arrest	0	0	0	
Discharged alive	6 (85.7)	0	6 (100)	
Post arrest CPC 1 and 2, no. (%)	1 (0.9)	0	1 (16.7)	0.054

^a^Shows a comparison between “Died” and “Survived”

^b^Shockable rhythm includes ventricular fibrillation, ventricular tachycardia, or unknown shockable rhythm

Abbreviations: *AED* automatic external defibrillation, *CPC* cerebral performance category, *CPR* cardiopulmonary resuscitation, *ED* emergency department, *EMS* emergency medical services, *ET* endotracheal tube, *LMA* laryngeal mask airway, *OHCA* out-of-hospital cardiac arrest, *ROSC* return of spontaneous circulation, *SD* standard deviation, *Time of the day* cardiac arrest occurred between 08:00 and 20:00

## Discussion

Of 111 patients with traumatic OHCA included in our analysis, nearly one fifth (18.0%) achieved ROSC at the scene of the cardiac arrest or on the way to hospital, only minority of patients survived to hospital admission (6.3%) and hospital discharge (5.4%), and survived with good neurological function (0.9%). We recognize that this cohort is likely to be highly selected as many patients with OHCA in Vietnam are not brought to hospital and might die outside of hospital [21, 25, 41]. In this study, the figure for the proportion of ROSC at the scene of the cardiac arrest or on the way to hospital is in line with the figure reported in our published previous study of patients with non-traumatic OHCA (19%; 112/590) [31]; however, our proportions for survival to hospital admission and survival to discharge from the hospital are lower than the rates reported in our previous study (24.2%; 143/590 and 14.1%, 83/590, respectively) [31]. A large multicenter, case-control study based on the French national cardiac arrest registry also shows that the rate of survival to hospital admission is lower in patients with traumatic OHCA (14%; 449/3209) than in patients with non-traumatic OHCA (20.4%; 8341/40,878) [5]. In the setting of traumatic cardiopulmonary arrest, the ACS COT guidelines state that outcome in cardiac arrest following trauma is dismal, especially in the cases of no obvious signs of life, injuries that are incompatible with life, evidence of prolonged arrest, and lack of organized electrocardiographic activity [6, 7].

In Vietnam, as well as in other LMICs, pre-hospital care and transportation systems are categorized into EMS and non-EMS (e.g., private ambulances, own or private transport, or public transport) [27, 34]. In our study, over two-fifths of the patients with traumatic OHCA were brought to the hospital by private or public transport without life-support equipment or trained personnel (Fig. 1 and Table 1). Pre-hospital care is, for the most part, left to bystanders, and usually, the injured or sick person is simply carried quickly to the nearest vehicle large enough to accommodate him or her [25, 30, 34]. In such situations, bystander first-aid is crucial to improve the outcomes of patients with trauma or traumatic OHCA [42]; bystander first-aid and chest compression, however, are not often done in Vietnam [30]. A previous study assessed exposure to severe bleeding, bleeding control knowledge, and willingness to intervene with and without trauma first-aid kits, and participants who received a trauma first-aid kit were significantly associated with increased post-training confidence [16]. Thus, to improve bystander first-aid, more lay people should be trained in first-aid and to be able to train others through a recognized trauma first-aid program [16, 43].

Along with economic and political reforms and motorization that have spurred rapid economic growth in Vietnam, [44] RTIs are becoming a major public health issue [19–23]. Policy changes are needed to mitigate this major public health issue. For example, injury prevention programs are effective in reducing RTIs: the helmet law enacted by the Vietnamese Government in December 2007 increased the incidence of helmet use among motorcyclists to around 85% and substantially decreased motorcycle-related head injuries (− 16%) and fatalities (− 18%) [45, 46]. However, healthcare providers still have difficulty in delivering essential initial care for patients with trauma or traumatic OHCA because of low resources and inadequate infrastructure for emergency medical care, such as dispatch centers for EMS [27, 29, 47].. In our study, a minority of patients with traumatic OHCA were attended to and taken to hospital by EMS (Table 1). Moreover, the proportions of patients who achieved ROSC at the scene or on the way to the hospital, who survived on hospital admission, and who survived to discharge from the hospital were lower in those taken to hospital by the EMS than those taken by private ambulance (Table 1). These findings might be explained that because of the small number of trained and qualified medical emergency staff and the limited amount of life-saving equipment, these staff are overworked and underequipped and the EMS centers are overburdened [27, 29]. These findings also might be attributed to private ambulances getting flagged down (they come across the call) instead of being dispatched, so there is less delay to care and shorter overall time to take patients to hospital. The present study shows that the prevalence of cardiac arrests witnessed by EMS was higher than those witnessed by private ambulances (Table 1). The time-stamped data on emergency calls at the dispatch center, EMS arrival on the scene, EMS departure from the scene, and arrival at the ED were often not available for non-EMS (i.e., private or public transport, private ambulance). Additionally, there are currently no criteria for calling EMS in Vietnam, and in almost 30% of calls that EMS responded to, the patients were no longer at the scene; they may have taken their own transport to the hospital [48, 49]. This suggests EMS may have selection bias for patients with the most serious illnesses or injuries. This also highlights lack of experience/practice among EMS and private ambulance clinicians due to low rates of utilization and increased scene time might be contributing to the negative findings.

Because of the limited pre-hospital care in Vietnam, in addition to a nationwide policy on the EMS system which has introduced in 2008, private ambulance services with the capability for first-aid, CPR, life-saving drugs, defibrillators or at least a medical professional trained to deal with emergencies have been established. In 2011, the health ministry gave these services licenses for first-aid or patient transportation and the policy has not changed since then [50]. However, the healthcare providers may not be sufficiently well trained or experienced to be able to provide first-aid in trauma, such as controlling life-threatening bleeding, providing intubation, needle aspiration, chest tube drainages, and thoracotomy. In our study, data on first-aid in trauma was not available; however, only a few patients with traumatic OHCA received pre-hospital advanced airway management by EMS or private ambulance services (Table 1). In Vietnam, the recruitment of new EMS personnel or healthcare providers is facing several challenges, [29] such as physicians and nurses, although, should undergo an 18-month clinical training program in inpatient settings after graduation to acquire their complete clinical license, [50] EMS is not considered an inpatient facility, which makes obtaining post-graduate training difficult [29]. Additionally, the lack of a trauma system of care prevents integration of pre-hospital and hospital treatment protocols [21, 25–29]. These factors might result in the low survival rates of patients with traumatic OHCA in our study.

In our study, only a small number of patients received administration of CPR by a bystander and resuscitation attempts by an EMS or a private ambulance (Table 1); proportions for both CPR by a bystander and resuscitation attempts by an EMS or a private ambulance, however, were significantly higher in patients who achieved ROSC than those who did not achieve ROSC at the scene of the cardiac arrest or on the way to the hospital (Table S2 as shown in Additional file 2). A multicenter, case-control study shows that the probability of survival, although, is lower for trauma victims, the efforts are not futile and pre-hospital resuscitation efforts seem worthwhile [5]. In our study, no significant difference between patients who died before hospital admission or hospital discharge and who survived to hospital admission or discharge from the hospital, however, was found for the administration of CPR by a bystander and resuscitation attempts by an EMS or a private ambulance (Table 2 and S8 as shown in Additional file 2). Along with the underdeveloped EMS system, the lack of an organized trauma system of care might result in the most common transportation method (approximately 50.7%) of patients with trauma in Vietnam was a motorbike, pre-hospital trauma teams mainly included emergency medical physicians and nurses, and the rate of deaths before reaching the hospital was higher than 50% [51]. These factors might prevent first-aid in trauma, resuscitation attempts, and post-resuscitation care.

Our study has some limitations. Our data are from a highly selected population of cases who were brought to the three highest-level public sector hospitals in Vietnam. Therefore, the number of patients with traumatic OHCA is likely to be considerably higher. In addition, data were missing for many variables, e.g., in only 30 patients was it recorded if the pre-hospital advanced airway management was given or not. Moreover, the limited pre-hospital data is available for cases brought by non-EMS (i.e., private or public transport, private ambulance). In our study, a significant proportion of patients with traumatic OHCA came to the hospital in private transport rather than by EMS or private ambulances. Some of these patients might be attended to by primary healthcare providers, may be pronounced dead at the scene of the cardiac arrest or might not be brought to the hospital at all. These factors resulted in incomplete enrolment of patients into the database of the study, which may have introduced selection bias [52]. These limitations might account for some differences in figures reported from other countries.

In conclusion, this was a highly selected cohort of patients with traumatic OHCA presenting to the ED with a low rate of EMS or private ambulance utilization and low survival rates. The low rate of EMS or private ambulance utilization and the poor survival emphasize the need for increasing bystander first-aid and developing an organized trauma system of care, increasing both the number of EMS ambulances and the use of private ambulances, and developing a standard emergency first-aid program for both healthcare personnel and the community.

## Supplementary Information

**Additional file 1.** Data collection form.

**Additional file 2.** Supplementary results.

**Additional file 3.** STROBE_checklist_cohort.

**Additional file 4.** Ethical Review Board Approval.

**Additional file 5.** Dataset.

## Data Availability

All data generated or analyzed during this study are included in this published article [and its supplementary information files].

## Abbreviations

ACLS: advanced cardiac life support
ACS COT: American College of Surgeons Committee on Trauma
AED: automated external defibrillation
BLS: basic life support
CI: confidence interval
CPC: cerebral performance category
CPR: cardiopulmonary resuscitation
ED: emergency department
EMS: emergency medical services
HIC: high-income country
IQR: interquartile range
LMIC: middle-income country
OHCA: out-of-hospital cardiac arrest
OR: odds ratio
PAROS: Pan-Asian Resuscitation Outcomes Study
ROSC: return of spontaneous circulation
RTI: road traffic injury
SD: standard deviation
