# Supplementary material for: Survival after traumatic out-of-hospital cardiac arrest in Vietnam: a multicenter prospective cohort study
Source: BMC Emerg Med. 2021 Nov 23;21:148. doi: 10.1186/s12873-021-00542-z (PMC8609736; doi:10.1186/s12873-021-00542-z)
Supplement: Supplementary file 1 — Additional file 1. Data collection form. [file 12873_2021_542_MOESM1_ESM.doc]

**Pan-Asian Resuscitation Outcomes Study (PAROS)**

|  |  | | | | | | | | | | | |
| --- | --- | --- | --- | --- | --- | --- | --- | --- | --- | --- | --- | --- |
|  | **Case number** |  |  |  |  |  |  |  |  |  |  |  |
|  |  | | | | | | | | | | | |

Mode of Transportation

|  |  | | | |
| --- | --- | --- | --- | --- |
| #1 | **Patient brought in by** | □_1_ EMS | □_2_ Non-EMS | |
|  | ***If ‘Non-EMS’, please specify*** | □*_1_* *Private ambulance* | □*_2_**Own/Private transport* | □*_3_* *Public transport* |
|  |  | | | |

Incident Information

|  | | | | | | | | | | |
| --- | --- | --- | --- | --- | --- | --- | --- | --- | --- | --- |
| #2 | **Date of incident** | | |  | | | (dd/mm/yyyy) | |  |  |
| #3 | **Location of incident** | | | | **(Optional)_________________________________________________________** | | | | | |
|  | **(enter Zip/Postal code)** | | | | _____________________________ □ *Unknown* | | | | | |
| #4 | **Location type** | □_1_ Home residence | | | | □_2_ Healthcare facility | | □_3_ Public/Commercial building | | |
|  |  | □_4_ Nursing home | | | | □_5_ Street/Highway | | □_6_ Industrial place | | |
|  |  | □_7_ Transport center | | | | □_8_ Place of recreation | | □_9_ In EMS/Private ambulance | | |
|  |  | □_10_ Other, specify ________________________________________________________ | | | | | | | | |
|  |  | |  | | | |  | | | |

Patient Information

|  | | | | | | | | | | | | | | | | | | | | | |
| --- | --- | --- | --- | --- | --- | --- | --- | --- | --- | --- | --- | --- | --- | --- | --- | --- | --- | --- | --- | --- | --- |
|  | | **Date of birth** | |  | | | | | (dd/mm/yyyy) | | | **Age** | | |  | | | | | | 🞏 Days |
| #5 |  | |  | | | | |  | | |  | | |  | | | | | | 🞏 Months | |
|  |  | |  | | | | |  | | |  | | |  | | | | | | 🞏 Years | |
| #6 | | **Gender** | | □_1_ Male | | □_2_ Female | | | | | | | | | | | | | | | |
| #7 | | **Race (optional)** | | □_1_ Chinese | □_2_ Malay | | | | | □_3_ Indian | | | □_4_ Eurasian | | | | | | □_5_ Other | | |
| #8 | | **Medical history** | | 🞅_1_ No | | | 🞅_2_ Unknown | | | | | | | | | | | 🞅_3_ Heart disease | | | |
|  |  | | 🞅_4_ Diabetes | | | 🞅_5_ Cancer | | | | | | | | | | 🞅_6_ Hypertension | | | | | |
|  | |  | | 🞅_7_ Renal disease | | | 🞅_8_ Respiratory disease | | | | | | | | | | 🞅_9_ Hyperlipidemia | | | | |
|  | |  | | 🞅_10_ Stroke | | | 🞅_11_ HIV | | | | | | | | | | 🞅_12_ Other | | | | |
|  | |  | | | | | | | | | | | | | | | | | | | |

Dispatch Information (Not Applicable for Non-EMS case)

|  | | | | |
| --- | --- | --- | --- | --- |
| #9 | **Time call received at dispatch center** |  | (hh:mm:ss) | □ ***No*** First Responder dispatched |
| #10 | **Time First responder dispatched** |  | (hh:mm:ss) |
| #11 | **Time Ambulance dispatched** |  | (hh:mm:ss) |
| #12 | **Time First responder arrived at scene time** |  | (hh:mm:ss) | |
| #13 | **Time Ambulance arrived at scene** |  | (hh:mm:ss) | |
| #14 | **Time EMS arrived at patient side** |  | (hh:mm:ss) | |
| #15 | **Time Ambulance left scene** |  | (hh:mm:ss) | |
| #16 | **Time Ambulance arrived at ED** |  | (hh:mm:ss) | |
|  |  | | | |

Prehospital Event and Resuscitation Information

|  |  | | | | | | | | | | | | | | | | | | | | | | |
| --- | --- | --- | --- | --- | --- | --- | --- | --- | --- | --- | --- | --- | --- | --- | --- | --- | --- | --- | --- | --- | --- | --- | --- |
| #17 | **Estimated time of arrest** | |  | | | | | | | | | (hh:mm:ss) | | | | | □ *Unknown* | | | | | | |
| #18 | **Arrest witnessed by** | | □_1_ Not witnessed | | | | | | | | | | | | | | | | | | | | |
|  |  | | □_2_ EMS/Private ambulance | | | | | | | | | | | | | | | | | | | | |
|  |  | | □_3_ Bystander – healthcare provider | | | | | | | | | | | | | | | | | | | | |
|  |  | | □_4_ Bystander – lay person | | | | | | | | | | | | | | | | | | | | |
|  |  | | □_5_ Bystander – family | | | | | | | | | | | | | | | | | | | | |
| #19 | **Bystander CPR** | | □_1_ Yes | | | | | | | □_2_ No | | | | | | | | | | | | | |
| #20 | ***First* CPR initiated by** | | □_1_ No CPR initiated | | | | | | | | | | | | | | | | | | | | |
|  |  | | □_2_ First responder | | | | | | | | | | | | | | | | | | | | |
|  |  | | □_3_ Ambulance crew | | | | | | | | | | | | | | | | | | | | |
|  |  | | □_4_ Bystander – healthcare provider | | | | | | | | | | | | | | | | | | | | |
|  |  | | □_5_ Bystander – lay person | | | | | | | | | | | | | | | | | | | | |
|  |  | | □_6_ Bystander – family | | | | | | | | | | | | | | | | | | | | |
|  |  | | □_7_ Unknown | | | | | | | | | | | | | | | | | | | | |
| #21 | **Bystander AED applied** | | □_1_ Yes | | | | | | | □_2_ No | | | | | | | | | | | | | |
| #22 | **Resuscitation attempted by EMS/Private ambulance** | | | | | | | | | | | | | | | □_1_ Yes | | | | | □_2_ No | | |
| #23 | **First arrest rhythm** | | | □_1_ VF | | | | □_2_ VT | | | | | | | | □_3_ PEA | | | | | □_4_ Asystole | | |
|  |  | | | □_5_ Unknown *shockable* rhythm | | | | | | | | | | | | | | | | | | | |
|  |  | | | □_6_ Unknown *unshockable* rhythm | | | | | | | | | | | | | | □_7_ Unknown | | | | | |
| #24 | **Time CPR started by EMS/Private ambulance** | | | | | | | | | |  | | | | | | | | | (hh:mm:ss) | | | □ *Unknown* |
| #25 | **Time AED applied by EMS/Private ambulance** | | | | | | | | | |  | | | | | | | | | (hh:mm:ss) | | | □ *Unknown* |
| #26 | **Prehospital defibrillation** | | | | □_1_ Yes | | | | | | □_2_ No | | | | | | | | | | | | |
|  | ***If ‘Yes’, time of first shock given*** | | | | | |  | | | | | | | *(hh:mm:ss)* | | | | | | □ *Unknown* | | | |
| #27 | | **Defibrillation performed by** | | | | 🞅_1_ First responder | | | | | | | | | | | | | | | | | |
|  | |  | | | | 🞅_2_ Ambulance crew | | | | | | | | | | | | | | | | | |
|  | |  | | | | 🞅_3_ Bystander – healthcare provider | | | | | | | | | | | | | | | | | |
|  | |  | | | | 🞅_4_ Bystander – lay person | | | | | | | | | | | | | | | | | |
|  | |  | | | | 🞅_5_ Bystander - family | | | | | | | | | | | | | | | | | |
| #28 | | **Mechanical CPR device used by EMS/Private ambulance** | | | | | | | | | | | | | □_1_ Yes | | | | | | | □_2_ No | |
|  | | ***If ‘Yes’, please specify*** | | | | | | | | | | | | | □*_1_ Load-Distributing Band* | | | | | | | | |
|  | |  | | | | | | | | | | | | | □*_2_ Active Compression Decompression* | | | | | | | | |
|  | |  | | | | | | | | | | | | | □*_3_ Mechanical Piston* | | | | | | | □*_4_ Other* | |
| #29 | | **Prehospital advanced airway** | | | | | | | | | | | | | □_1_ Yes | | | | | | | □_2_ No | |
|  | | ***If ‘Yes’, please specify*** | | | | | | | | | | | | | □*_1_ Oral/Nasal ET* | | | | | | | □*_4_ King airway* | |
|  | |  | | | | | | | | | | | | | □*_2_ Combitube* | | | | | | | □*_5_ Other* | |
|  | |  | | | | | | | | | | | | | □*_3_ LMA* | | | | | | | | |
| #30 | | **Prehospital drug administration** | | | | | | | | | | | | | □_1_ Yes | | | | | | | □_2_ No | |
|  | | ***If ‘Yes’, select drugs given*** | | | | | | | | | | | | | 🞅*_1_ Epinephrine* | | | | | | | 🞅*_5_ Lidocaine* | |
|  | |  | | | | | | | | | | | | | 🞅*_2_ Atropine* | | | | | | | 🞅*_6_ Dextrose* | |
|  | |  | | | | | | | | | | | | | 🞅*_3_ Amiodarone* | | | | | | | 🞅*_7_ Other* | |
|  | |  | | | | | | | | | | | | | 🞅*_4_ Bicarbonate* | | | | | | |  | |
| #31 | | **Return of spontaneous circulation at scene/en-route** | | | | | | | | | | | | | □_1_ Yes | | | | | | | □_2_ No | |
|  | | ***If ‘Yes’, specify time*** | | | | | | |  | | | | | | | | | | *(hh:mm:ss)* | | | □ *Unknown* | |
| #32 | | **CPR discontinued at scene/en-route** | | | | | | | | | | | | | □_1_ Yes | | | | | | | □_2_ No | |
|  | | ***If ‘Yes’, please specify*** | | | | | | | | | | | □_1_ *DNAR* | | | | | | | | | | |
|  | |  | | | | | | | | | | | □*_2_ ROSC* | | | | | | | | | | |
|  | |  | | | | | | | | | | | □*_3_ Medical control order* | | | | | | | | | | |
|  | |  | | | | | | | | | | | □*_4_ Obvious signs of death* | | | | | | | | | | |
|  | |  | | | | | | | | | | | □*_5_ Protocol/policy requirements completed* | | | | | | | | | | |
|  | |  | | | | | | | | | | | | | | | | | | | | | |

Disposition

|  |  | | | | | |
| --- | --- | --- | --- | --- | --- | --- |
| #33 | **Final status at scene** | □_1_ Conveyed to ED | | | □_2_ Pronounced dead at scene | |
| #34 | **Cause of arrest** | □_1_Trauma | | | □_2_ Non-trauma | |
|  | ***If ‘Non-trauma’, please specify*** | □_1_ *Presumed cardiac etiology* | | | □*_2_ Respiratory* | |
|  |  | □*_3_ Electrocution* | | □*_4_ Drowning* | □*_5_ Other* | |
| #35 | **Level of destination hospital** | □_1_ Tertiary | | | □_2_ Community | |
| #36 | **Destination hospital** | □_1_ AH | □_2_ CGH | | □_3_ KKH | □_4_ KTPH |
|  |  | □_5_ NUH | □_6_ TTSH | | □_7_ SGH | □_8_ N.A. |
| #37 | **Patient’s status at ED arrival** | □_1_ ROSC | | | | |
|  |  | □_2_ Ongoing resuscitation | | | | |
|  |  | □_3_ Transported without resuscitation | | | | |
|  |  | | | | | |

ED Resuscitation Information (Not Applicable for cases that were pronounced dead at scene)

|  |  | | | | | | | | | | | | | | | |
| --- | --- | --- | --- | --- | --- | --- | --- | --- | --- | --- | --- | --- | --- | --- | --- | --- |
| #38 | **Date of arrival at ED** | |  | | | | | | | | (dd/mm/yyyy) | | | | | |
| #39 | **Time of arrival at ED** | |  | | | | | (hh:mm:ss) | | | | | | | | |
| #40 | **Patient status on arrival at ED** | | Pulse | | | | □_1_ Yes | | | □_2_ No | | | | | | |
|  |  | | *Breathing* | | | | □_1_ Yes | | | □_2_ No | | | | | | |
| #41 | **Cardiac rhythm on arrival at ED** | | | | | | □_1_ VF | | | □_2_ VT | | | | □_3_ PEA | | |
|  |  | | | | | | □_4_ Asystole | | □_5_ Sinus or other perfusing rhythm | | | | | | | |
| #42 | **ED defibrillation performed** | | | | | | □_1_ Yes | | | □_2_ No | | | | | | |
| #43 | **Mechanical CPR device used at ED** | | | | □_1_ Yes | | | | | | | □_2_ No | | | | |
|  | ***If ‘Yes’, please specify*** | | | | □*_1_ Load-Distributing Band* | | | | | | | | | | | |
|  |  | | | | □*_2_ Active Compression Decompression* | | | | | | | | | | | |
|  |  | | | | □*_3_ Mechanical Piston* | | | | | | | □*_4_ Other* | | | | |
| #44 | **Advanced airway used at ED** | | | | □_1_ Yes | | | | | | | □_2_ No | | | | |
|  | ***If ‘Yes’, please specify*** | | | | □*_1_ Oral/Nasal ET* | | | | | | | □*_4_ King airway* | | | | |
|  |  | | | | □*_2_ Combitube* | | | | | | | □*_5_ Other* | | | | |
|  |  | | | | □*_3_ LMA* | | | | | | | | | | | |
| #45 | **Drug administered at ED** | | | | □_1_Yes | | | | | | | □_2_ No | | | | |
|  | ***If ‘Yes’, select drugs given*** | | | | 🞅*_1_ Epinephrine* | | | | | | | 🞅*_5_ Lidocaine* | | | | |
|  |  | | | | 🞅*_2_ Atropine* | | | | | | | 🞅*_6_ Dextrose* | | | | |
|  |  | | | | 🞅*_3_ Amiodarone* | | | | | | | 🞅*_7_ Other* | | | | |
|  |  | | | | 🞅*_4_ Bicarbonate* | | | | | | |  | | | | |
| #46 | **Return of spontaneous circulation at ED** | | | | □_1_ Yes | | | | | | | □_2_ No | | | | □_3_ NA |
|  | ***If ‘Yes’, specify time*** | | | |  | | | | | | | | *(hh:mm:ss)* | | □ *Unknown* | |
| #47 | **Emergency PCI performed** | | | | □_1_ Yes | | | | | | | □_2_ No | | | | |
| #48 | **Emergency CABG performed** | | | | □_1_ Yes | | | | | | | □_2_ No | | | | |
| #49 | **Hypothermia therapy initiated** | | | | □_1_ Yes | | | | | | | □_2_ No | | | | |
| #50 | **ECMO therapy initiated** | | | | □_1_ Yes | | | | | | | □_2_ No | | | | |
| #51 | **Cause of arrest** | | | | □_1_ Trauma | | | | | | | □_2_ Non-trauma | | | | |
|  | ***If ‘Non-trauma’, please specify*** | | | | □_1_ *Presumed cardiac etiology* | | | | | | | | | | □*_2_ Respiratory* | |
|  |  | | | | □*_3_ Electrocution* | | | | | | | □*_4_ Drowning* | | | □*_5_ Other* | |
| #52 | **Reason for discontinuing CPR at ED** | | | | □_1_ Death | | | | | | | □_3_ ROSC | | | | |
|  |  | | | | | □_2_ DNAR | | | | | | □_4_ ECMO therapy | | | | |
| #53 | **Outcome of patient** | □_1_ Admitted | | | | | | | | | | □_3_ Died in ED | | | | |
|  |  | □_2_ Transferred to another hospital | | | | | | | | | | □_4_ Unknown | | | | |
|  |  | | |  | | | | | | | | | | | | |

Hospital Outcome *(FOR PATIENT WHO SURVIVED TO ADMISSION)*

|  |  | | | |
| --- | --- | --- | --- | --- |
| #54 | **Patient status** | □_1_ Discharged alive | | |
|  |  | □_2_ Remains in hospital at 30^th^ day post arrest | | |
|  |  | □_3_ Died in hospital | | |
| #55 | **Date of Discharge or Death** |  | (dd/mm/yyyy) | |
| #56 | **Patient neurological status on discharge or at 30^th^ day post arrest** | Cerebral Performance Category | |  |
|  |  | Overall Performance Category | |  |
|  |  |  | | □ Unknown |
|  |  | | | |

Patient Health and Quality of Life □ **Unknown**

*(FOR PATIENT WHO IS DISCHARGED ALIVE or ALIVE ON 30^th^ DAY POST ARREST)*

|  |  | |  | | | | |
| --- | --- | --- | --- | --- | --- | --- | --- |
|  | **EQ-5D Health Dimensions** | | | |  | | |
| #57 | Mobility | □_1_ No problem | | □_2_ Some problems | | □_3_ Confined to bed | |
| #58 | Self-care | □_1_ No problem | | □_2_ Some problems | | □_3_ Unable to wash or dress | |
| #59 | Usual activities | □_1_ No problem | | □_2_ Some problems | | □_3_ Unable to perform | |
| #60 | Pain/Discomfort | □_1_ None | | □_2_ Moderate | | □_3_ Extreme | |
| #61 | Anxiety/Depression | □_1_ None | | □_2_ Moderate | | □_3_ Extreme | |
|  |  | | | | | | |
| #62 | **EQ-5D Visual Analog Scale (VAS)** | | | | | | |
|  | 100  90  80  70  60  50  40  30  20  10  0 | | | | | | |
|  | **100 (best imaginable health state) and 0 (worst imaginable health state)* | | | | | | |
|  |  | |  | | | |  |
