# Supplementary material for: Survival after traumatic out-of-hospital cardiac arrest in Vietnam: a multicenter prospective cohort study
Source: BMC Emerg Med. 2021 Nov 23;21:148. doi: 10.1186/s12873-021-00542-z (PMC8609736; doi:10.1186/s12873-021-00542-z)
Supplement: Supplementary file 2 — Additional file 2. Supplementary results. [file 12873_2021_542_MOESM2_ESM.docx]

**SUPPLEMENTAL RESULTS**

**Table S1.** General characteristics, pre- and in-hospital management, and outcomes of patients with out-of-hospital cardiac arrest according to types of pre-hospital transportation

| Characteristics | All cases  (n=111) | Non-EMS | | EMS  (n= 29) | p-value^*^ |
| --- | --- | --- | --- | --- | --- |
|  |  | Private or public transport  (n= 48) | Private ambulance  (n= 34) |  |  |
| **Hospital participated** | n=111 | n=48 | n=34 | n=29 | <0.001 |
| Bach Mai hospital, no. (%) | 33 (29.7) | 13 (27.1) | 19 (55.9) | 1 (3.4) |  |
| Hue hospital, no. (%) | 26 (23.4) | 21 (43.8) | 3 (8.8) | 2 (6.9) |  |
| Cho Ray hospital, no. (%) | 52 (46.8) | 14 (29.2) | 12 (35.3) | 26 (89.7) |  |
| **Patient related** | n=111 | n=48 | n=34 | n=29 |  |
| Age (year), mean (SD) | 39.27 (16.38) | 38.31 (14.74) | 40.71 (17.59) | 39.17 (17.91) | 0.931 |
| Gender, no. (%) | n=111 | n=48 | n=34 | n=29 | 0.993 |
| Male | 92 (82.9) | 40 (83.3) | 28 (82.4) | 24 (82.8) |  |
| Female | 19 (17.1) | 8 (16.7) | 6 (17.6) | 5 (17.2) |  |
| Past medical history, no. (%) | n=67 | n=23 | n=27 | n=17 |  |
| Heart disease | 2 (3.0) | 1 (4.3) | 1 (3.7) | 0 | >0.999 |
| Diabetes | 2 (3.0) | 0 | 2 (7.4) | 0 | 0.335 |
| Cancer | 0 | 0 | 0 | 0 |  |
| Hypertension | 6 (9.0) | 2 (8.7) | 4 (14.8) | 0 | 0.268 |
| Renal disease | 2 (3.0) | 1 (4.3) | 0 | 1 (5.9) | 0.512 |
| Respiratory disease | 1 (1.5) | 1 (4.3) | 0 | 0 | 0.597 |
| Hyperlipidemia | 0 | 0 | 0 | 0 |  |
| Stroke | 0 | 0 | 0 | 0 |  |
| HIV | 0 | 0 | 0 | 0 |  |
| Other | 9 (13.4) | 2 (8.7) | 7 (25.9) | 0 | 0.039 |
| **Event related** |  |  |  |  |  |
| Location type, no. (%) | n=111 | n=48 | n=34 | n=29 | 0.001 |
| Home residence | 20 (18.0) | 11 (22.9) | 8 (23.5) | 1 (3.4) |  |
| Healthcare facility | 4 (3.6) | 0 | 4 (11.8) | 0 |  |
| In EMS/Private ambulance | 6 (5.4) | 0 | 5 (14.7) | 1 (3.4) |  |
| Industrial place | 8 (7.2) | 2 (4.2) | 2 (5.9) | 4 (13.8) |  |
| Nursing home | 0 | 0 | 0 | 0 |  |
| Place of recreation | 0 | 0 | 0 | 0 |  |
| Public/Commercial building | 0 | 0 | 0 | 0 |  |
| Street/Highway | 69 (62.2) | 32 (66.7) | 14 (41.2) | 23 (79.3) |  |
| Transport center | 4 (0.9) | 0 | 1 (2.9) | 0 |  |
| Other | 3 (2.7) | 3 (6.3) | 0 | 0 |  |
| Time of the day, no. (%) | 34/60 (56.7) | 6/11 (54.5) | 15/23 (65.2) | 13/26 (50.0) | 0.556 |
| Arrest witnessed by, no. (%) | n=93 | n=30 | n=34 | n=29 | <0.001 |
| Not witnessed | 22 (23.7) | 14 (46.7) | 7 (20.6) | 1 (3.4) |  |
| Bystander (Lay person) | 10 (10.8) | 8 (26.7) | 1 (2.9) | 1 (3.4) |  |
| Bystander (Family) | 5 (5.4) | 0 | 4 (11.8) | 1 (3.4) |  |
| Bystander (Healthcare provider) | 14 (15.0) | 8 (26.7) | 5 (14.7) | 1 (3.4) |  |
| EMS/Private ambulance | 42 (45.2) | 0 | 17 (50.0) | 25 (86.2) |  |
| First arrest rhythm, no. (%) | n=30 | - | n=21 | n=9 | 0.182 |
| VT | 2 (6.7) | not available | 0 | 2 (22.2) |  |
| VF | 2 (6.7) | not available | 2 (9.5) | 0 |  |
| Unknown shockable rhythm | 15 (50.0) | not available | 12 (57.1) | 3 (33.3) |  |
| Unknown unshockable rhythm | 0 | not available | 0 | 0 |  |
| PEA | 9 (30.0) | not available | 6 (28.6) | 3 (33.3) |  |
| Asystole | 2 (6.7) | not available | 1 (4.8) | 1 (11.1) |  |
| First arrest rhythm, no. (%) |  |  |  |  | 0.687 |
| Shockable rhythm | 19 (63,3) | not available | 14 (66,7) | 5 (55,6) |  |
| Unshockable rhythm | 11 (36,7) | not available | 7 (33,3) | 4 (44,4) |  |
| Prehospital intervention, no. (%) |  |  |  |  |  |
| Bystander CPR | 32/95 (33.7) | 0/32 | 19/34 (55.9) | 13/29 (44.8) | <0.001 |
| Prehospital defibrillation, | 6/30 (20) | not available | 3/21 (14.3) | 3/9 (33.3) | 0.329 |
| Bystander AED applied, | 2 (1.8) | 0 | 1 (2.9) | 1/19 (3.4) | 0.320 |
| ED defibrillation performed, no. (%) | 6/111 (5.4) | 1/48 (2.1) | 4/34 (11.8) | 1/29 (3.4) | 0.200 |
| **System related** |  |  |  |  |  |
| Resuscitation attempted by EMS/private ambulance, no. (%) | 29/53 (54.7) | not available | 20/34 (58.8) | 9/19 (47.4) | 0.422 |
| Time to CPR at scene (min), n=20, mean (SD) | 2.68 (5.66) | not available | 2.53 (4.74) | 2.95 (7.52) | 0.553 |
| **Therapeutic related** |  |  |  |  |  |
| Pharmacotherapy, no. (%) | n=111 | n=48 | n=34 | n=29 |  |
| Epinephrine (at scene) | 27 (24.3) | 0 | 19 (55.9) | 8 (27.6) | <0.001 |
| Epinephrine (at ED) | 101 (91.0) | 44 (91.7) | 30 (88.2) | 27 (93.1) | 0.837 |
| Prehospital advanced airway, no. (%) | 27/30 (90.0) | not available | 18/21 (85.7) | 9/9 (100) | 0.534 |
| Prehospital advanced airway techniques, no. (%) | n=27 | - | n=18 | n=9 | 0.333 |
| Oral/Nasal ET | 26 (96.3) | not available | 18 (100) | 8 (88.9) |  |
| LMA | 1 (3.7) | not available | 0 | 1 (11.1) |  |
| Other |  |  |  |  |  |
| Advanced airway used at ED, no. (%) | 65/111 (58.6) | 35/48 (72.9) | 12/34 (35.3) | 18/29 (62.1) | 0.003 |
| Advanced airway techniques used at ED, *n (%)* | n=64 | n=35 | n=12 | n=17 | - |
| Oral/Nasal ET | 64 (100) | 35 (100) | 12 (100) | 17 (100) |  |
| LMA | 0 | 0 | 0 | 0 |  |
| Other | 0 | 0 | 0 | 0 |  |
| Immediate coronary angiography on admission to hospital, no. (%), |  |  |  |  |  |
| Emergency PCI performed | 0 | 0 | 0 | 0 |  |
| Emergency CABG performed | 0 | 0 | 0 | 0 |  |
| Post-resuscitation care, no. (%) |  |  |  |  |  |
| ECMO therapy initiated, | 0 | 0 | 0 | 0 |  |
| Hypothermia therapy initiated | 2 (1.8) | 0 | 2 (5.9) | 0 | 0.158 |
| **Outcomes** |  |  |  |  |  |
| ROSC, no. (%) | n=111 | n=48 | n=34 | n=29 |  |
| ROSC at scene/en-route, | 20 (18.0) | 0 | 14 (41.2) | 6 (20.7) | <0.001 |
| ROSC at ED, | 16 (14.4) | 6 (12.5) | 5 (14.7) | 5 (17.2) | 0.889 |
| Cumulative ROSC | 33 (29.7) | 6 (12.5) | 17 (50.0) | 10 (34.5) |  |
| Outcome of patient at ED, no. (%) | n=111 | n=48 | n=34 | n=29 | 0.197 |
| Died in ED | 104 (93.7) | 47 (97.9) | 30 (88.2) | 27 (93.1) |  |
| Admitted | 7 (6.3) | 1 (2.1) | 4 (11.8) | 2 (6.9) |  |
| Patient status, no. (%) | n=7 | n=1 | n=4 | n=2 | 0.429 |
| Died in the hospital | 1 (14.3) | 0 | 0 | 1 (50.0) |  |
| Remains in hospital at 30th day post arrest | 0 | 0 | 0 | 0 |  |
| Discharged alive | 6 (85.7) | 1 (100) | 4 (100) | 1 (50.0) |  |
| Post arrest CPC 1 and 2, no. (%) | 1 (0.9) | 0 | 1 (2.9) | 0 | 0.568 |
| ^*^ Shows comparison between "EMS", "Private ambulance" and "Private or public transport".  **AED**, automatic external defibrillation; **CABG**, coronary artery bypass grafting; **CPC**, cerebral performance category; **CPR**, cardiopulmonary resuscitation; **ECMO**, extracorporeal membrane oxygenation; **ED**, emergency department; **EMS**, emergency medical services; **ET**, endotracheal; **LMA**, laryngeal mask airway; **OHCA**, out-of-hospital cardiac arrest; **PCI**, percutaneous coronary intervention; **PEA**, pulseless electrical activity; **ROSC**, return of spontaneous circulation; **SD**, standard deviation; **Time of the day**, period from 8:00 to 20:00 hour; **VF**, ventricular fibrillation; **VT**, ventricular tachycardia. | | | | | |

**Table S2.** General characteristics, pre- and in-hospital management, and outcomes of patients with out-of-hospital cardiac arrest according to return of spontaneous circulation at scene/en-route

| Characteristics | All cases  (n=111) | ROSC at Scene  (n= 20) | Not ROSC at Scene  (n=91) | p-value^*^ |
| --- | --- | --- | --- | --- |
| **Hospital participated** | | | | |
| Hospital | n=111 | n=20 | n=91 | 0.020 |
| Bach Mai hospital, no. (%) | 33 (29.7) | 11 (55.0) | 22 (24.2) |  |
| Hue hospital, no. (%) | 26 (23.4) | 2 (10.0) | 24 (26.4) |  |
| Cho Ray hospital, no. (%) | 52 (46.8) | 7 (35.0) | 45 (49.5) |  |
| **Patient related** | | | | |
| Age, mean (SD) | 39.27 (16.38) | 38.25 (15.67) | 43.90 (19.06) | 0.267 |
| Gender, no. (%) | n=111 | n=20 | n=91 | 0.517 |
| Male | 92 (82.9) | 18 (90.0) | 74 (81.3) |  |
| Female | 19 (17.1) | 2 (10.0) | 17 (18.7) |  |
| Past medical history, no. (%) | n=67 | n=13 | n=54 |  |
| Heart disease | 2 (3.0) | 0 | 2 (3.7) | >0.999 |
| Diabetes | 2 (3.0) | 0 | 2 (3.7) | >0.999 |
| Cancer | 0 | 0 | 0 |  |
| Hypertension | 6 (9.0) | 1 (7.7) | 5 (9.3) | >0.999 |
| Renal disease | 2. (3.0) | 2 (3.0) | 1 (7.7) | 0.353 |
| Respiratory disease | 1 (1.5) | 0 | 1 (1.9) | >0.999 |
| Hyperlipidemia | 0 | 0 | 0 |  |
| Stroke | 0 | 0 | 0 |  |
| HIV | 0 | 0 | 0 |  |
| Other | 9 (13.4) | 5 (38.5) | 4 (7.4) | 0.011 |
| **Event related** | | | | |
| Location type, no. (%) | n=111 | n=20 | n=91 | 0.020 |
| Home residence | 20 (18.0) | 8 (40.0) | 12 (13.2) |  |
| Healthcare facility | 4 (3.6) | 1 (5.0) | 3 (3.3) |  |
| In EMS/Private ambulance | 6 (5.4) | 0 | 6 (6.6) |  |
| Industrial place | 8 (7.2) | 2 (10.0) | 6 (6.6) |  |
| Nursing home | 0 | 0 | 0 |  |
| Place of recreation | 0 | 0 | 0 |  |
| Public/Commercial building | 0 | 0 | 0 |  |
| Street/Highway | 69 (62.2) | 8 (40.0) | 61 (67.0) |  |
| Transport center | 1 (0.9) | 1 (5.0) | 0 |  |
| Other | 3 (2.7) | 0 | 3 (3.7) |  |
| Time of the day, no. (%) | 34/60 (56.7) | 8/14 (57.1) | 26/46 (56.5) | 0.967 |
| Arrest witnessed by, no. (%) | n=93 | n=20 | n=73 | 0.743 |
| Not witnessed | 22 (23.7) | 4 (20.0) | 18 (24.7) |  |
| Bystander (Lay person) | 10 (10.8) | 2 (10.0) | 8 (11.0) |  |
| Bystander (Family) | 5 (5.4) | 1 (5.0) | 4 (5.5) |  |
| Bystander (Healthcare provider) | 14 (15.1) | 5 (25.0) | 9 (12.3) |  |
| EMS/Private ambulance | 42 (45.2) | 8 (40.0) | 34 (46.6) |  |
| First arrest rhythm, no. (%) | n=30 | n=20 | n=10 | 0.072 |
| VT | 2 (6.7) | 2 (10.0) | 0 |  |
| VF | 2 (6.7) | 2 (10.0) | 0 |  |
| Unknown shockable rhythm | 15 (50.0) | 12 (60.0) | 3 (30.0) |  |
| Unknown unshockable rhythm | 0 | 0 | 0 |  |
| PEA | 9 (30.0) | 3 (15.0) | 6 (60.0) |  |
| Asystole | 2 (6.7) | 1 (5.0) | 1 (10.0) |  |
| First arrest rhythm, no. (%) | n=30 | n=20 | n=10 | 0.015 |
| Shockable rhythm | 19 (63.3) | 16 (80.0) | 3 (30.0) |  |
| Unshockable rhythm | 11 (36.7) | 4 (20.0) | 7 (70.0) |  |
| Prehospital intervention, no. (%) |  |  |  |  |
| Bystander CPR | 32/95 (33.7) | 11/20 (55.0) | 21/75 (28.0) | 0.023 |
| Prehospital defibrillation, | 6/30 (20.0) | 5/20 (25.0) | 1/10 (10.0) | 0.633 |
| Bystander AED applied, | 2 (1.8) | 0 | 2(2.2) | >0.999 |
| ED defibrillation performed, no. (%) | 6/111 (5.4) | 2/20 (10.0) | 4/91 (4.4) | 0.295 |
| **System related** | | | | |
| Types of prehospital transportation, no. (%) | n=111 | n=20 | n=91 | <0.001 |
| Private or public transport | 48 (43.2) | 0 | 48 (52.7) |  |
| Private ambulance | 34 (30.6) | 14 (70.0) | 20 (22.0) |  |
| EMS | 29 (26.1) | 6 (30.0) | 23 (25.3) |  |
| Resuscitation attempted by EMS/private ambulance, no. (%) | 29/53 (54.7) | 20/20 (100) | 9/33 (27.3) | <0.001 |
| Time to CPR at scene (min), n=20, mean (SD) | 2.68 (5.66) | 2.32 (6.63) | 2.97 (5.06) | 0.194 |
| **Therapeutic related** | | | | |
| Pharmacotherapy, no. (%) | n=111 | n=20 | n=91 |  |
| Epinephrine (at scene) | 27 (24.3) | 19 (95.0) | 8 (8.8) | <0.001 |
| Epinephrine (at ED) | 101 (91.0) | 16 (80.0) | 85 (93.4) | 0.079 |
| Prehospital advanced airway, no. (%) | 27/30 (90.0) | 20/20 (100) | 7/10 (70.0) | 0.030 |
| Prehospital advanced airway techniques, no. (%) | n=27 | n=20 | n=7 | >0.999 |
| Oral/Nasal ET | 26 (96.3) | 19 (95.0) | 7 (100) |  |
| LMA | 1 (3.7) | 1 (5.0) | 0 |  |
| Other | 0 | 0 | 0 |  |
| Advanced airway used at ED, no. (%) | 65/111 (58.6) | 0/20 | 65/91 (71.4) | <0.001 |
| Immediate coronary angiography on admission to hospital, no. (%) | n=111 | n=20 | n=91 |  |
| Emergency PCI performed | 0 | 0 | 0 |  |
| Emergency CABG performed | 0 | 0 | 0 |  |
| Post-resuscitation care, no. (%) | n=111 | n=20 | n=91 |  |
| ECMO therapy initiated, | 0 | 0 | 0 |  |
| Hypothermia therapy initiated | 2 (1.8) | 2 (10.0) | 0 | 0.031 |
| **Outcomes** | | | | |
| ROSC, no. (%) | n=111 | n=20 | n=91 |  |
| ROSC at ED, | 16 (14.4) | 3 (15.0) | 13 (14.3) | >0.999 |
| Cumulative ROSC | 33 (29.7) | 20 (100) | 13 (14.3) |  |
| Outcome of patient at ED, no. (%) | n=111 | n=20 | n=91 | 0.109 |
| Died in ED | 104 (93.7) | 17 (85.0) | 87 (95.6) |  |
| Admitted | 7 (6.3) | 3 (15.0) | 4 (4.4) |  |
| Patient status, no. (%) | n=7 | n=3 | n=4 | 0.429 |
| Died in the hospital | 1 (14.3) | 1 (33.3) | 0 |  |
| Remains in hospital at 30th day post arrest | 0 | 0 | 0 |  |
| Discharged alive | 6 (85.7) | 2 (66.7) | 4 (100) |  |
| Post arrest CPC 1 and 2, no. (%) | 1/111 (0.9) | 0/20 | 1/91 (1.1) | >0.999 |

**Table S3.** Factors relating to ROSC at scene/en-route: A univariate analysis

| Factors | Frequency | OR | 95.0% CI for OR | | p-value |
| --- | --- | --- | --- | --- | --- |
|  |  |  | Lower | Upper |  |
| **Hospital participated** |  |  |  |  |  |
| Hospital |  |  |  |  |  |
| Bach Mai hospital, | 33 | - | - | - | 0.029 |
| Hue hospital, | 26 | 0.167 | 0.033 | 0.837 | **0.030** |
| Cho Ray hospital, | 52 | 0.311 | 0.106 | 0.913 | **0.033** |
| **Patient related** |  |  |  |  |  |
| Age (year) | 111 | 1.020 | 0.992 | 1.050 | 0.166 |
| Gender |  |  |  |  |  |
| Male | 92 | 2.068 | 0.437 | 9.771 | 0.359 |
| **Past medical history** |  |  |  |  |  |
| Heart disease | 2 | 0.000 | 0.000 | - | 0.999 |
| Diabetes | 2 | 0.000 | 0.000 | - | 0.999 |
| Hypertension | 6 | 0.817 | 0.087 | 7.655 | 0.859 |
| Renal disease | 2 | 4.417 | 0.258 | 75.727 | 0.306 |
| Respiratory disease | 1 | 0.000 | 0.000 | - | >0.999 |
| Other | 9 | 7.812 | 1.723 | 35.430 | 0.008 |
| **Event related** | | | | | |
| Location type: |  |  |  |  |  |
| Home residence | 20 | 4.389 | 1.489 | 12.940 | 0.007 |
| Street/Highway |  |  |  |  |  |
| Time of the day: |  |  |  |  |  |
| Period from 8:00 to 20:00 hour | 34 | 1.026 | 0.306 | 3.434 | 0.967 |
| Witness status: |  |  |  |  |  |
| Not witnessed | 22 | - | - | - | 0.752 |
| Bystander (Lay person) | 10 | 1.125 | 0.170 | 7.452 | 0.903 |
| Bystander (Family) | 5 | 1.125 | 0.098 | 12.965 | 0.925 |
| Bystander (Healthcare provider) | 14 | 2.500 | 0.536 | 11.651 | 0.243 |
| EMS/Private ambulance | 42 | 1.059 | 0.280 | 4.001 | 0.933 |
| First arrest rhythm: |  |  |  |  |  |
| VT/VF/ Unknown Shockable Rhythm | 19 | 9.333 | 1.637 | 53.208 | **0.012** |
| Prehospital intervention: |  |  |  |  |  |
| Bystander CPR | 32 | 3.143 | 1.139 | 8.672 | **0.027** |
| Prehospital defibrillation | 6 | 3.000 | 0.301 | 29.940 | 0.349 |
| Bystander AED applied | 2 | 0.000 | 0.000 | - | 0.999 |
| ED defibrillation performed | 6 | 2.417 | 0.411 | 14.211 | 0.329 |
| **System related** | | | | | |
| Types of prehospital transportation, no. (%) |  |  |  |  |  |
| Private or public transport | 48 | - | - | - | 0.230 |
| Private ambulance | 34 | 1130832139 | 0.000 | - | 0.997 |
| EMS | 29 | 421428126.2 | 0.000 | - | 0.997 |
| Resuscitation attempted by EMS/Private ambulance | 29 | 3589945322 | 0.000 | - | 0.998 |
| Time to CPR (at scene) | 20 | 1.022 | 0.868 | 1.203 | 0.796 |
| **Therapeutic related** | | | | | |
| Pharmacotherapy: |  |  |  |  |  |
| Epinephrine (at scene) | 27 | 197.125 | 23.244 | 1671.737 | **<0.001** |
| Epinephrine (at ED) | 101 | 0.282 | 0.072 | 1.115 | 0.071 |
| Advanced airway management: |  |  |  |  |  |
| Prehospital advanced airway | 27 | 4615643589 | 0.000 | - | 0.999 |
| Advanced airway used at ED | 65 | 0.000 | 0.000 | - | 0.997 |
| Post-resuscitation care |  |  |  |  |  |
| Hypothermia therapy initiated | 2 | 8167122925 | 0.000 | - | 0.999 |
| Outcome of patient at ED |  |  |  |  |  |
| Died in ED | 104 | - | - | - | - |
| Admitted | 7 | 3.838 | 0.787 | 18.720 | 0.096 |
| Patient status, |  |  |  |  |  |
| Died in the hospital | 1 | - | - | - | - |
| Discharged alive | 6 | 0.000 | 0.000 | - | >0.999 |
| Post arrest CPC 1 and 2 | 1 | 0.000 | 0.000 | - | >0.999 |

**Table S4.** Factors relating to ROSC at scene/en-route: A multivariate analysis

| Steps | Factors | Unit | OR | 95.0% CI for OR | | p-value |
| --- | --- | --- | --- | --- | --- | --- |
|  |  |  |  | Lower | Upper |  |
| 1 | Age Group (≥69) | % | 2.601 | 0.117 | 57.961 | 0.546 |
|  | Home residence | % | 23464233268812620 | 0.000 | - | 0.998 |
|  | Street/Highway | % | 1.591 | 0.183 | 13.854 | 0.674 |
|  | VT/VF/ Unknown Shockable Rhythm | % | 6.682 | 0.748 | 59.717 | 0.089 |
|  | Bystander CPR | % | 1.098 | 0.089 | 13.621 | 0.942 |
|  | Prehospital defibrillation | % | 1.566 | 0.090 | 27.287 | 0.758 |
|  | Epinephrine (at scene) | % | 0.000 | 0.000 | - | 0.999 |
|  | Constant |  | 0.352 |  |  | 0.504 |
| 2 | Age Group (≥69) | % | 3.337 | 0.150 | 74.430 | 0.447 |
|  | Home residence | % | 619979978.008 | 0.000 | - | 0.999 |
|  | Street/Highway | % | 1.968 | 0.246 | 15.734 | 0.523 |
|  | VT/VF/ Unknown Shockable Rhythm | % | 5.874 | 0.720 | 47.960 | 0.098 |
|  | Bystander CPR | % | 0.942 | 0.079 | 11.187 | 0.962 |
|  | Prehospital defibrillation | % | 2.122 | 0.137 | 32.760 | 0.590 |
|  | Constant |  | 0.264 |  |  | 0.394 |
| 3 | Age Group (≥69) | % | 3.224 | 0.179 | 58.167 | 0.428 |
|  | Street/Highway | % | 1.187 | 0.148 | 9.484 | 0.872 |
|  | VT/VF/ Unknown Shockable Rhythm | % | 9.628 | 1.159 | 80.005 | 0.036 |
|  | Bystander CPR | % | 0.522 | 0.054 | 5.045 | 0.574 |
|  | Prehospital defibrillation | % | 1.916 | 0.127 | 28.992 | 0.6399 |
|  | Constant |  | 0.593 |  |  | 0.712 |
| 4 | Age Group (≥69) | % | 3.147 | 0.178 | 55.644 | 0.434 |
|  | VT/VF/ Unknown Shockable Rhythm | % | 8.875 | 1.396 | 56.422 | 0.021 |
|  | Bystander CPR | % | 0.528 | 0.055 | 5.044 | 0.579 |
|  | Prehospital defibrillation | % | 1.992 | 0.138 | 28.755 | 0.613 |
|  | Constant |  | 0.668 |  |  | 0.737 |
| 5 | Age Group (≥69) | % | 2.584 | 0.155 | 43.031 | 0.508 |
|  | VT/VF/ Unknown Shockable Rhythm | % | 9.675 | 1.555 | 60.196 | 0.015 |
|  | Bystander CPR | % | 0.436 | 0.052 | 3.675 | 0.445 |
|  | Constant |  | 0.855 |  |  | 0.887 |
| 6 | VT/VF/ Unknown Shockable Rhythm | % | 8.959 | 1.403 | 53.405 | 0.016 |
|  | Bystander CPR | % | 0.333 | 0.045 | 2.443 | 0.280 |
|  | Constant |  | 1.240 |  |  | 0.822 |
| 7 | VT/VF/ Unknown Shockable Rhythm | % | 9.333 | 1.637 | 53.208 | 0.012 |
|  | Constant |  | 0.571 |  |  | 0.372 |
| **CI**, confidence interval; **ED**, emergency department; **EMS**, emergency medical services; **OR**, odds ratio; **ROSC**, return of spontaneous circulation; **Time of the day**, period from 8:00 to 20:00 hour. | | | | | | |

**Table S5.** General characteristics, pre- and in-hospital management, and outcomes of patients with out-of-hospital cardiac arrest according to return of spontaneous circulation at scene/en-route or in the ED

| Characteristics | All cases  (n=111) | Not ROSC  (n= 78) | ROSC  (n=33) | p-value^*^ |
| --- | --- | --- | --- | --- |
| **Hospital participated** | | | | |
| Hospital | n=111 | n=78 | n=33 | 0.274 |
| Bach Mai hospital, no. (%) | 33 (29.7) | 20 (25.6) | 13 (39.4) |  |
| Hue hospital, no. (%) | 26 (23.4) | 18 (23.1) | 8 (24.2) |  |
| Cho Ray hospital, no. (%) | 52 (46.8) | 40 (51.3) | 12 (36.4) |  |
| **Patient related** | | | | |
| Age (year), mean (SD) | 39.27 (16.38) | 39.58 (15.89) | 38.55 (17.72) | 0.500 |
| Gender, no. (%) | n=111 | n=78 | n=33 | 0.721 |
| Male | 92 (82.9) | 64 (82.1) | 28 (84.8) |  |
| Female | 19 (17.1) | 14 (17.9) | 5 (15.2) |  |
| Past medical history, no. (%) | n=67 | n=47 | n=20 |  |
| Heart disease | 2 (3.0) | 2 (4.3) | 0 | >0.999 |
| Diabetes | 2 (3.0) | 2 (4.3) | 0 | >0.999 |
| Hypertension | 6 (9.0) | 4 (8.5) | 2 (10.0) | >0.999 |
| Renal disease | 2. (3.0) | 1 (2.1) | 1 (5.0) | 0.511 |
| Respiratory disease | 1 (1.5) | 1 (2.1) | 0 | >0.999 |
| Other | 9 (13.4) | 2 (4.3) | 7 (35.0) | 0.002 |
| **Event related** | | | | |
| Location type, no. (%) | n=111 | n=78 | n=33 | 0.209 |
| Home residence | 20 (18.0) | 11 (14.1) | 9 (27.3) |  |
| Healthcare facility | 4 (3.6) | 2 (2.6) | 2 (6.1) |  |
| In EMS/Private ambulance | 6 (5.4) | 5 (6.4) | 1 (3.0) |  |
| Industrial place | 8 (7.2) | 5 (6.4) | 3 (9.1) |  |
| Street/Highway | 69 (62.2) | 52 (66.7) | 17 (51.5) |  |
| Transport center | 1 (0.9) | 0 | 1 (3.0) |  |
| Other | 3 (2.7) | 3 (3.8) | 0 |  |
| Time of the day, no. (%) | 34/60 (56.7) | 24/40 (60.0) | 10/20 (50.0) | 0.461 |
| Arrest witnessed by, no. (%) | n=93 | n=65 | n=28 | 0.454 |
| Not witnessed | 22 (23.7) | 17 (26.2) | 5 (17.9) |  |
| Bystander (Lay person) | 10 (10.8) | 8 (12.3) | 2 (7.1) |  |
| Bystander (Family) | 5 (5.4) | 4 (6.2) | 1 (3.6) |  |
| Bystander (Healthcare provider) | 14 (15.1) | 7 (10.8) | 7 (25.0) |  |
| EMS/Private ambulance | 42 (45.2) | 29 (44.6) | 13 (46.4) |  |
| First arrest rhythm, no. (%) | n=30 | n=9 | n=21 | **0.037** |
| VT | 2 (6.7) | 0 | 2 (9.5) |  |
| VF | 2 (6.7) | 0 | 2 (9.5) |  |
| Unknown shockable rhythm | 15 (50.0) | 2 (22.2) | 13 (61.9) |  |
| Unknown unshockable rhythm | 0 | 0 | 0 |  |
| PEA | 9 (30.0) | 6 (66.7) | 3 (14.3) |  |
| Asystole | 2 (6.7) | 1 (11.1) | 1 (4.8) |  |
| First arrest rhythm, no. (%) | n=30 | n=9 | n=21 | 0.004 |
| Shockable rhythm | 19 (63.3) | 2 (22.2) | 17 (81.0) |  |
| Unshockable rhythm | 11 (36.7) | 7 (77.8) | 4 (19.0) |  |
| Prehospital intervention, no. (%) |  |  |  |  |
| Bystander CPR | 32/95 (33.7) | 19/67 (28.4) | 13/28 (46.4) | 0.089 |
| Prehospital defibrillation, | 6/30 (20.0) | 1/9 (11.1) | 5/21 (23.8) | 0.637 |
| Bystander AED applied, | 2 (1.8) | 1 (1.3) | 1 (3.0) | 0.508 |
| ED defibrillation performed, no. (%) | 6/111 (5.4) | 2/78 (2.6) | 4/33 (12.1) | 0.063 |
| **System related** | | | | |
| Types of prehospital transportation, no. (%) | n=111 | n=78 | n=33 | **0.001** |
| Private or public transport | 48 (43.2) | 42 (53.8) | 6 (18.2) |  |
| Private ambulance | 34 (30.6) | 17 (21.8) | 17 (51.5) |  |
| EMS | 29 (26.1) | 19 (24.4) | 10 (30.3) |  |
| Resuscitation attempted by EMS/private ambulance, no. (%) | 29/53 (54.7) | 9/27 (33.3) | 20/26 (76.9) | **0.001** |
| Time to CPR at scene (min), n=20, mean (SD) | 2.68 (5.66) | 2.32 (6.63) | 2.97 (5.06) | 0.194 |
| **Therapeutic related** | | | | |
| Pharmacotherapy, no. (%) | n=111 | n=78 | n=33 |  |
| Epinephrine (at scene) | 27 (24.3) | 7 (9.0) | 20 (60.6) | **<0.001** |
| Epinephrine (at ED) | 101 (91.0) | 72 (92.3) | 29 (87.9) | 0.480 |
| Prehospital advanced airway, no. (%) | 27/30 (90.0) | 7/9 (77.8) | 20/21 (95.2) | 0.207 |
| Prehospital advanced airway techniques, no. (%) | n=27 | n=7 | n=20 | >0.999 |
| Oral/Nasal ET | 26 (96.3) | 7 (100) | 19 (95.0) |  |
| LMA | 1 (3.7) | 0 | 1 (5.0) |  |
| Other | 0 | 0 | 0 |  |
| Advanced airway used at ED, no. (%) | 65/111 (58.6) | 52/78 (66.7) | 13/33 (39.4) | **0.008** |
| Post-resuscitation care, no. (%) | n=111 | n=78 | n=33 |  |
| Hypothermia therapy initiated | 2 (1.8) | 0 | 2 (6.1) | 0.086 |
| **Outcomes** | | | | |
| Outcome of patient at ED, no. (%) | n=111 | n=78 | n=33 | 0.024 |
| Died in ED | 104 (93.7) | 76 (97.4) | 28 (84.8) |  |
| Admitted | 7 (6.3) | 2 (2.6) | 5 (15.2) |  |
| Patient status, no. (%) | n=7 | n=2 | n=5 | >0.999 |
| Died in the hospital | 1 (14.3) | 0 | 1 (20.0) |  |
| Remains in hospital at 30th day post arrest | 0 | 0 | 0 |  |
| Discharged alive | 6 (85.7) | 2 (100) | 4 (80.0) |  |
| Post arrest CPC 1 and 2, no. (%) | 1/111 (0.9) | 0/78 | 1/33 (3.0) | 0.297 |

**Table S6.** Factors relating to cumulative ROSC at scene/en-route or in the ED: A univariate analysis

| Factors | Frequency | OR | 95.0% CI for OR | | p-value |
| --- | --- | --- | --- | --- | --- |
|  |  |  | Lower | Upper |  |
| **Hospital participated** |  |  |  |  |  |
| Hospital |  |  |  |  |  |
| Bach Mai hospital, | 33 | - | - | - | 0.280 |
| Hue hospital, | 26 | 0.684 | 0.231 | 2.027 | 0.493 |
| Cho Ray hospital, | 52 | 0.462 | 0.178 | 1.194 | 0.111 |
| **Patient related** |  |  |  |  |  |
| Age (year) | 111 | 0.996 | 0.971 | 1.022 | 0.761 |
| Gender |  |  |  |  |  |
| Male | 92 | 1.225 | 0.402 | 3.730 | 0.721 |
| **Past medical history** |  |  |  |  |  |
| Heart disease | 2 | 0.000 | 0.000 | - | 0.999 |
| Diabetes | 2 | 0.000 | 0.000 | - | 0.999 |
| Hypertension | 6 | 1.194 | 0.201 | 7.114 | 0.845 |
| Renal disease | 2 | 2.421 | 0.144 | 40.734 | 0.539 |
| Respiratory disease | 1 | 0.000 | 0.000 | - | >0.999 |
| Other | 9 | 12.115 | 2.239 | 65.548 | 0.004 |
| **Event related** | | | | | |
| Location type: |  |  |  |  |  |
| Home residence | 20 | 2.284 | 0.843 | 6.188 | 0.104 |
| Street/Highway | 69 | 0.531 | 0.232 | 1.217 | 0.135 |
| Time of the day: |  |  |  |  |  |
| Period from 8:00 to 20:00 hour | 34 | 0.667 | 0.226 | 1.965 | 0.462 |
| Witness status: |  |  |  |  |  |
| Not witnessed | 22 | - | - | - | 0.440 |
| Bystander (Lay person) | 10 | 0.850 | 0.135 | 5.366 | 0.863 |
| Bystander (Family) | 5 | 0.850 | 0.077 | 9.440 | 0.895 |
| Bystander (Healthcare provider) | 14 | 3.400 | 0.800 | 14.441 | 0.097 |
| EMS/Private ambulance | 42 | 1.524 | 0.462 | 5.023 | 0.489 |
| First arrest rhythm: |  |  |  |  |  |
| VT/VF/ Unknown shockable Rhythm | 19 | 14.875 | 2.198 | 100.656 | **0.006** |
| Prehospital intervention: |  |  |  |  |  |
| Bystander CPR | 32 | 2.189 | 0.879 | 5.456 | 0.093 |
| Prehospital defibrillation | 6 | 2.500 | 0.248 | 25.153 | 0.437 |
| Bystander AED applied | 2 | 2.406 | 0.146 | 39.661 | 0.539 |
| ED defibrillation performed | 6 | 5.241 | 0.910 | 30.176 | 0.064 |
| **System related** | | | | | |
| Types of prehospital transportation: |  |  |  |  |  |
| Private or public transport | 48 | - | - | - | 0.002 |
| Private ambulance | 34 | 7.000 | 2.358 | 20.777 | **<0.001** |
| EMS | 29 | 3.684 | 1.169 | 11.613 | **0.026** |
| Resuscitation attempted by EMS/Private ambulance | 29 | 6.667 | 1.981 | 22.435 | **0.002** |
| Time to CPR (at scene) | 20 | 1.022 | 0.868 | 1.203 | 0.796 |
| **Therapeutic related** | | | | | |
| Pharmacotherapy: |  |  |  |  |  |
| Epinephrine (at scene) | 27 | 15.604 | 5.492 | 44.336 | **<0.001** |
| Epinephrine (at ED) | 101 | 1.655 | 0.435 | 6.300 | 0.460 |
| Advanced airway management: |  |  |  |  |  |
| Prehospital advanced airway | 27 | 5.714 | 0.446 | 73.191 | 0.180 |
| Advanced airway used at ED | 65 | 0.325 | 0.140 | 0.754 | **0.009** |
| Post-resuscitation care |  |  |  |  |  |
| Hypothermia therapy initiated | 2 | 4064743207 | 0.000 | - | 0.999 |
| Outcome of patient at ED |  |  |  |  |  |
| Died in ED | 104 | - | - | - | - |
| Admitted | 7 | 6.786 | 1.244 | 37.000 | 0.027 |
| Patient status, |  |  |  |  |  |
| Died in the hospital | 1 | - | - | - | - |
| Discharged alive | 6 | 0.000 | 0.000 | - | >0.999 |
| Post arrest CPC 1 and 2 | 1 | 3937719982 | 0.000 | - | >0.999 |

**Table S7.** Factors relating to cumulative ROSC: A multivariate analysis

| Steps | Factors | Unit | OR | 95.0% CI for OR | | p-value |
| --- | --- | --- | --- | --- | --- | --- |
|  |  |  |  | Lower | Upper |  |
| 1 | Age group (≥60) | % | 3.906 | 0.085 | 179.202 | 0.485 |
|  | Home residence | % | 96322746722616816 | 0.000 | - | 0.998 |
|  | Street/Highway | % | 1.346 | 0.108 | 16.840 | 0.818 |
|  | VT/VF/ Unknown Shockable Rhythm | % | 13.649 | 0.718 | 259.385 | 0.082 |
|  | Bystander CPR | % | 5.346 | 0.023 | 1258.142 | 0.547 |
|  | Prehospital defibrillation | % | 0.895 | 0.018 | 44.716 | 0.956 |
|  | ED defibrillation performed | % | 0.284 | 0.005 | 14.654 | 0.531 |
|  | Resuscitation attempted by EMS/Private ambulance | % | 0.000 | 0.000 |  | 0.999 |
|  | Epinephrine (at scene) | % | 1382863759.678 | 0.000 |  | 0.998 |
|  | Advanced airway used at ED | % | 0.000 | 0.000 |  | >0.999 |
|  | Constant |  | 68304500.870 |  |  | >0.999 |
| 2 | Age group (≥60) | % | 4.101 | 0.086 | 195.379 | 0.474 |
|  | Home residence | % | 75777808381223024 | 0.000 | - | 0.998 |
|  | Street/Highway | % | 1.058 | 0.088 | 12.751 | 0.964 |
|  | VT/VF/ Unknown Shockable Rhythm | % | 15.235 | 0.866 | 267.914 | 0.063 |
|  | Bystander CPR | % | 4.437 | 0.021 | 937.164 | 0.585 |
|  | Prehospital defibrillation | % | 0.988 | 0.019 | 50.542 | 0.995 |
|  | ED defibrillation performed | % | 0.347 | 0.007 | 17.988 | 0.599 |
|  | EMS | % | 0.327 | 0.002 | 54.287 | 0.668 |
|  | Resuscitation attempted by EMS/Private ambulance |  | 0.000 | 0.000 |  |  |
|  | Epinephrine (at scene) |  | 1262494217.600 | 0.000 |  |  |
|  | Constant |  | 0.058 |  |  | >0.999 |
| 3 | Age group (≥60) | % | 4.253 | 0.082 | 220.733 |  |
|  | Home residence | % | 74642396530485072 | 0.000 | - | 0.4736 |
|  | Street/Highway | % | 0.989 | 0.083 | 11.830 | 0.998 |
|  | VT/VF/ Unknown Shockable Rhythm | % | 17.508 | 1.004 | 305.422 | 0.993 |
|  | Bystander CPR | % | 4.610 | 0.017 | 1267.742 | 0.050 |
|  | Prehospital defibrillation | % | 0.993 | 0.018 | 55.189 | 0.594 |
|  | ED defibrillation performed | % | 0.307 | 0.006 | 16.744 | 0.997 |
|  | EMS | % | 0.339 | 0.002 | 72.349 | 0.563 |
|  | Epinephrine (at scene) |  | 1493633428.869 | 0.000 | - | 0.693 |
|  | Constant |  | 0.000 |  |  | 0.998 |
| 4 | Age group (≥60) | % | 3.823 | 0.097 | 151.338 | 0.475 |
|  | Home residence | % | 300645988.735 | 0.000 | - | 0.999 |
|  | Street/Highway | % | 1.295 | 0.143 | 11.757 | 0.818 |
|  | VT/VF/ Unknown Shockable Rhythm | % | 9.544 | 0.970 | 93.869 | 0.053 |
|  | Bystander CPR | % | 1.241 | 0.043 | 35.970 | 0.900 |
|  | Prehospital defibrillation | % | 1.723 | 0.069 | 43.307 | 0.741 |
|  | ED defibrillation performed | % | 0.553 | 0.017 | 18.103 | 0.739 |
|  | EMS | % | 0.959 | 0.047 | 19.504 | 0.978 |
|  | Constant |  | 0.322 |  |  | 0.515 |
| 5 | Age group (≥60) | % | 4.770 | 0.116 | 195.728 | 0.410 |
|  | Street/Highway | % | 1.090 | 0.107 | 11.143 | 0.942 |
|  | VT/VF/ Unknown Shockable Rhythm | % | 15.421 | 1.497 | 158.912 | 0.022 |
|  | Bystander CPR | % | 0.633 | 0.031 | 12.765 | 0.765 |
|  | Prehospital defibrillation | % | 2.088 | 0.094 | 46.436 | 0.642 |
|  | ED defibrillation performed | % | 0.342 | 0.009 | 12.449 | 0.558 |
|  | EMS | % | 1.928 | 0.113 | 32.876 | 0.650 |
|  | Constant |  | 0.374 |  |  | 0.582 |
| 6 | Age group (≥60) | % | 4.616 | 0.126 | 169.584 | 0.406 |
|  | VT/VF/ Unknown Shockable Rhythm | % | 14.838 | 1.868 | 117.839 | 0.011 |
|  | Bystander CPR | % | 0.641 | 0.033 | 12.642 | 0.770 |
|  | Prehospital defibrillation | % | 2.104 | 0.095 | 46.530 | 0.638 |
|  | ED defibrillation performed | % | 0.353 | 0.011 | 11.576 | 0.559 |
|  | EMS | % | 1.865 | 0.127 | 27.347 | 0.649 |
|  | Constant |  | 0.404 |  |  | 0.529 |
| 7 | Age group (≥60) | % | 5.711 | 0.220 | 148.557 | 0.295 |
|  | VT/VF/ Unknown Shockable Rhythm | % | 15.180 | 1.929 | 119.436 | 0.010 |
|  | Prehospital defibrillation | % | 2.363 | 0.116 | 48.030 | 0.576 |
|  | ED defibrillation performed | % | 0.302 | 0.011 | 8.050 | 0.475 |
|  | EMS | % | 1.585 | 0.142 | 17.659 | 0.708 |
|  | Constant |  | 0.314 |  |  | 0.316 |
| 8 | Age group (≥60) | % | 4.775 | 0.221 | 103.358 | 0.319 |
|  | VT/VF/ Unknown Shockable Rhythm | % | 15.655 | 2.020 | 121.306 | 0.008 |
|  | Prehospital defibrillation | % | 1.904 | 0.123 | 29.486 | 0.645 |
|  | ED defibrillation performed | % | 0.384 | 0.019 | 7.821 | 0.533 |
|  | Constant |  | 0.436 |  |  | 0.253 |
| 9 | Age group (≥60) | % | 4.160 | 0.204 | 84.834 | 0.354 |
|  | VT/VF/ Unknown Shockable Rhythm | % | 17.136 | 2.254 | 130.276 | 0.006 |
|  | ED defibrillation performed | % | 0.422 | 0.022 | 8.174 | 0.569 |
|  | Constant |  | 0.472 |  |  | 0.284 |
| 10 | Age group (≥60) | % | 3.089 | 0.213 | 44.768 | 0.408 |
|  | VT/VF/ Unknown Shockable Rhythm | % | 15.726 | 2.218 | 111.510 | 0.006 |
|  | Constant |  | 0.458 |  |  | 0.263 |
| 11 | VT/VF/ Unknown Shockable Rhythm | % | 14.875 | 2.198 | 100.656 | 0.006 |
|  | Constant |  | 0.571 |  |  | 0.372 |
| **CI**, confidence interval; **ED**, emergency department; **EMS**, emergency medical services; **OR**, odds ratio; **ROSC**, return of spontaneous circulation; **Time of the day**, period from 8:00 to 20:00 hour. | | | | | | |

**Table S8.** Characteristics, management and outcomes of patients with out-of-hospital cardiac arrest according to survival to hospital admission, Viet Nam, February 2014 - December 2018

| Characteristics | All cases  (n=111) | Died  (n= 104) | Survived  (n=7) | p-value^*^ |
| --- | --- | --- | --- | --- |
| **Hospital participated** | | | | |
| Hospital | n=111 | n=104 | n=7 | 0.779 |
| Bach Mai hospital, no. (%) | 33 (29.7) | 30 (28.8) | 3 (42.9) |  |
| Hue hospital, no. (%) | 26 (23.4) | 25 (24.0) | 1 (14.3) |  |
| Cho Ray hospital, no. (%) | 52 (46.8) | 49 (47.1) | 3 (42.9) |  |
| **Patient related** | | | | |
| Age (year), mean (SD) | 39.27 (16.38) | 39.17 (16.40) | 40.71 (17.40) | 0.785 |
| Gender, no. (%) |  |  |  | >0.999 |
| Male | 92 (82.9) | 86 (82.7) | 6 (85.7) |  |
| Female | 19 (17.1) | 18 (17.3) | 1 (14.3) |  |
| Past medical history, no. (%) | n=67 | n=61 | n=6 |  |
| Heart disease | 2 (3.0) | 2 (3.3) | 0 | >0.999 |
| Diabetes | 2 (3.0) | 2 (3.3) | 0 | >0.999 |
| Hypertension | 6 (9.0) | 4 (6.6) | 2 (33.3) | 0.086 |
| Renal disease | 2 (3.0) | 2 (3.3) | 0 | >0.999 |
| Respiratory disease | 1(1.5) | 1 (1.6) | 0 | >0.999 |
| Other | 9 (13.4) | 7 (11.5) | 2 (33.3) | 0.181 |
| **Event related** | | | | |
| Location type, no. (%) | n=111 | n=104 | n=7 | 0.280 |
| Home residence | 20 (18.0) | 17 (16.3) | 3 (42.9) |  |
| Healthcare facility | 4 (3.6) | 3 (2.9) | 1 (14.3) |  |
| In EMS/Private ambulance | 6 (5.4) | 6 (5.8) | 0 |  |
| Industrial place | 8 (7.2) | 8 (7.7) | 0 |  |
| Street/Highway | 69 (62.2) | 66 (63.5) | 3 (42.9) |  |
| Transport center | 1 (0.9) | 1 (1.0) | 0 |  |
| Other | 3 (2.7) | 3 (2.9) | 0 |  |
| Time of the day, n=60, no. (%) | 34/60 (56.7) | 31/56 (55.4) | 3/4 (75.0) | 0.626 |
| Arrest witnessed by, no. (%) | n=93 | n=86 | n=7 | 0.424 |
| Not witnessed | 22 (23.7) | 21 (24.4) | 1 (14.3) |  |
| Bystander (Lay person) | 10 (10.8) | 10 (11.6) | 0 |  |
| Bystander (Family) | 5 (5.4) | 4 (4.7) | 1 (14.3) |  |
| Bystander (Healthcare provider) | 14 (15.1) | 12 (14.0) | 2 (28.6) |  |
| EMS/Private ambulance | 42 (45.2) | 39 (45.3) | 3 (42.9) |  |
| First arrest rhythm, no. (%) | n=30 | n=27 | n=3 | 0.094 |
| VT | 2 (6.7) | 1 (3.7) | 1 (33.3) |  |
| VF | 2 (6.7) | 1 (3.7) | 1 (33.3) |  |
| Unknown shockable rhythm | 15 (50.0) | 14 (51.9) | 1 (33.3) |  |
| Unknown unshockable rhythm | 0 | 0 | 0 |  |
| PEA | 9 (30.0) | 9 (33.3) | 0 |  |
| Asystole | 2 (6.7) | 2 (7.4) | 0 |  |
| First arrest rhythm, no. (%) | n=30 | n=27 | n=3 | 0.279 |
| Shockable rhythm | 19 (63.3) | 16 (59.3) | 3 (100) |  |
| Unshockable rhythm | 11 (36.7) | 11 (40.7) | 0 |  |
| Prehospital intervention, no. (%) |  |  |  |  |
| Bystander CPR | 32/95 (33.7) | 29/88 (33.0) | 3/7 (42.9) | 0.684 |
| Prehospital defibrillation, | 6/30 (20.0) | 4/27 (14.8) | 2/3 (66.7) | 0.094 |
| Bystander AED applied, | 2/111 (1.8) | 2/104 (1.9) | 0/7 | >0.999 |
| ED defibrillation performed, no. (%) | 6/111(5.4) | 6/104 (5.8) | 0/7 | >0.999 |
| **System related** | | | | |
| Types of prehospital transportation, no. (%) | n=111 | n=104 | n=7 | 0.197 |
| Private or public transport | 48 (43.2) | 47 (45.2) | 1 (14.3) |  |
| Private ambulance | 34 (30.6) | 30 (28.8) | 4 (57.1) |  |
| EMS | 29 (26.1) | 27 (26.0) | 2 (28.6) |  |
| Resuscitation attempted by EMS/private ambulance, no. (%) | 29/53 (54.7) | 26/47 (55.3) | 3/6 (50.0) | >0.999 |
| Time to CPR at scene | n=20 | n=18 | n=2 |  |
| Time to CPR at scene (min), mean (SD) | 2.68 (5.66) | 2.13 (5.12) | 7.56 (10.52) | 0.225 |
| **Therapeutic related** | | | | |
| Pharmacotherapy, no. (%) | n=111 | n=104 | n=7 |  |
| Epinephrine (at scene) | 27 (24.3) | 24 (23.1) | 3 (42.9) | 0.358 |
| Epinephrine (at ED) | 101 (91.0) | 96 (92.3) | 5 (71.4) | 0.120 |
| Prehospital advanced airway, no. (%) | 27/30 (90.0) | 24/27 (88.9) | 3/3 (100) | >0.999 |
| Prehospital advanced airway techniques, no. (%) | n=27 | n=24 | n=3 | >0.999 |
| Oral/Nasal ET | 26 (96.3 ) | 23 (95.8) | 3 (100) |  |
| LMA | 1 (3.7) | 1 (4.2) | 0 |  |
| Other | 0 | 0 | 0 |  |
| Advanced airway used at ED, no. (%) | 65/111 (58.6) | 62/104 (59.6) | 3/7 (42.9) | 0.446 |
| Post-resuscitation care, no. (%) | n=111 | n=104 | n=7 |  |
| Hypothermia therapy initiated | 2 (1.8) | 0 | 2 (28.6) | 0.003 |
| **Outcomes** | | | | |
| Patient status, no. (%) | n=7 | n=0 | n=7 | - |
| Died in the hospital | 1 (14.3) | - | 1 (14.3) |  |
| Remains in hospital at 30th day post arrest | 0 | - | 0 |  |
| Discharged alive | 6 (85.7) | - | 6 (85.7) |  |
| Post arrest CPC 1 and 2, no. (%) | 1/111 (0.9) | 0/104 | 1/7 (14.3) | 0.063 |

**Table S9.** Characteristics, management and outcomes of patients with out-of-hospital cardiac arrest according to survival to hospital discharge, Viet Nam, February 2014 - December 2018

| Characteristics | All cases  (n=111) | Died  (n= 105) | Survived  (n=6) | p-value^*^ |
| --- | --- | --- | --- | --- |
| **Hospital participated** | | | | |
| Hospital | n=111 | n=105 | n=6 | 0.558 |
| Bach Mai hospital, no. (%) | 33 (29.7) | 30 (28.6) | 3 (50.0) |  |
| Hue hospital, no. (%) | 26 (23.4) | 25 (23.8) | 1 (16.7) |  |
| Cho Ray hospital, no. (%) | 52 (46.8) | 50 (47.6) | 2 (33.3) |  |
| **Patient related** | | | | |
| Age (year), mean (SD) | 39.27 (16.38) | 39.0 (16.41) | 44.00 (16.51) | 0.379 |
| Gender, no. (%) |  |  |  | >0.999 |
| Male | 92 (82.9) | 58 (82.9) | 5 (83.3) |  |
| Female | 19 (17.1) | 18 (17.1) | 1 (16.7) |  |
| Past medical history, no. (%) | n=67 | n=61 | n=6 |  |
| Heart disease | 2 (3.0) | 2 (3.3) | 0 | >0.999 |
| Diabetes | 2 (3.0) | 2 (3.3) | 0 | >0.999 |
| Hypertension | 6 (9.0) | 4 (6.6) | 2 (33.3) | 0.086 |
| Renal disease | 2 (3.0) | 2 (3.3) | 0 | >0.999 |
| Respiratory disease | 1(1.5) | 1 (1.6) | 0 | >0.999 |
| Other | 9 (13.4) | 7 (11.5) | 2 (33.3) | 0.181 |
| **Event related** | | | | |
| Location type, no. (%) | n=111 | n=105 | n=6 | 0.171 |
| Home residence | 20 (18.0) | 17 (16.2) | 3 (50.0) |  |
| Healthcare facility | 4 (3.6) | 3 (2.9) | 1 (16.7) |  |
| In EMS/Private ambulance | 6 (5.4) | 6 (5.7) | 0 |  |
| Industrial place | 8 (7.2) | 8 (7.6) | 0 |  |
| Street/Highway | 69 (62.2) | 67 (63.8) | 2 (33.3) |  |
| Transport center | 1 (0.9) | 1 (1.0) | 0 |  |
| Other | 3 (2.7) | 3 (2.9) | 0 |  |
| Time of the day, n=60, no. (%) | 34/60 (56.7) | 31/57 (54.4) | 3/3 (100) | 0.251 |
| Arrest witnessed by, no. (%) | n=93 | n=87 | n=6 | 0.305 |
| Not witnessed | 22 (23.7) | 21 (24.1) | 1 (16.7) |  |
| Bystander (Lay person) | 10 (10.8) | 10 (11.5) | 0 |  |
| Bystander (Family) | 5 (5.4) | 4 (4.6) | 1 (16.7) |  |
| Bystander (Healthcare provider) | 14 (15.1) | 12 (13.8) | 2 (33.3) |  |
| EMS/Private ambulance | 42 (45.2) | 40 (46.0) | 2 (33.3) |  |
| First arrest rhythm, no. (%) | n=30 | n=28 | n=2 | 0.366 |
| VT | 2 (6.7) | 2 (7.1) | 0 |  |
| VF | 2 (6.7) | 1 (3.6) | 1 (50.0) |  |
| Unknown shockable rhythm | 15 (50.0) | 14 (50.0) | 1 (50.0) |  |
| Unknown unshockable rhythm | 0 | 0 | 0 |  |
| PEA | 9 (30.0) | 9 (32.1) | 0 |  |
| Asystole | 2 (6.7) | 2 (7.1) | 0 |  |
| First arrest rhythm, no. (%) | n=30 | n=28 | n=2 | 0.520 |
| Shockable rhythm | 19 (63.3) | 17 (60.7) | 2 (100) |  |
| Unshockable rhythm | 11 (36.7) | 11 (39.3) | 0 |  |
| Prehospital intervention, no. (%) |  |  |  |  |
| Bystander CPR | 32/95 (33.7) | 30/89 (33.7) | 2/6 (33.3) | >0.999 |
| Prehospital defibrillation, | 6/30 (20.0) | 5/28 (17.9) | 1/2 (50.0) | 0.366 |
| Bystander AED applied, | 2/111 (1.8) | 2/105 (1.9) | 0 | >0.999 |
| ED defibrillation performed, no. (%) | 6/111(5.4) | 6/105 (5.7) | 0 | >0.999 |
| **System related** | | | | |
| Types of prehospital transportation, no. (%) | n=111 | n=105 | n=6 | 0.200 |
| Private or public transport | 48 (43.2) | 47 (44.8) | 1 (16.7) |  |
| Private ambulance | 34 (30.6) | 30 (28.6) | 4 (66.7) |  |
| EMS | 29 (26.1) | 28 (26.7) | 1 (16.7) |  |
| Resuscitation attempted by EMS/private ambulance, no. (%) | 29/53 (54.7) | 27/48 (56.3) | 2/5 (40.0) | 0.649 |
| Time to CPR at scene | n=20 | n=19 | n=1 |  |
| Time to CPR at scene (min), mean (SD) | 2.68 (5.66) | 2.03 (4.99) | 15 | 0.115 |
| **Therapeutic related** | | | | |
| Pharmacotherapy, no. (%) | n=111 | n=105 | n=6 |  |
| Epinephrine (at scene) | 27 (24.3) | 25 (23.8) | 2 (33.3) | 0.632 |
| Epinephrine (at ED) | 101 (91.0) | 96 (91.4) | 5 (83.3) | 0.440 |
| Prehospital advanced airway, no. (%) | 27/30 (90.0) | 25/28 (89.3) | 2/2 (100) | >0.999 |
| Prehospital advanced airway techniques, no. (%) | n=27 | n=25 | n=2 | >0.999 |
| Oral/Nasal ET | 26 (96.3 ) | 24 (96.0) | 2 (100) |  |
| LMA | 1 (3.7) | 1 (4.0) | 0 |  |
| Other | 0 | 0 | 0 |  |
| Advanced airway used at ED, no. (%) | 65/111 (58.6) | 62/105 (59.0) | 3/6 (50.0) | 0.691 |
| Post-resuscitation care, no. (%) | n=111 | n=105 | n=6 |  |
| Hypothermia therapy initiated | 2 (1.8) | 0 | 2 (33.3) | 0.002 |
| **Outcomes** | | | | |
| Outcome of patient at ED, no. (%) | n=111 | n=105 | n=6 | <0.001 |
| Died in ED | 104 (93.7) | 104 (99.0) | 0 |  |
| Admitted | 7 (6.3) | 1 (1.0) | 6 (100) |  |
| Patient status, no. (%) | n=7 | n=1 | n=6 | 0.143 |
| Died in the hospital | 1 (14.3) | 1 (100) | 0 |  |
| Remains in hospital at 30th day post arrest | 0 | 0 | 0 |  |
| Discharged alive | 6 (85.7) | 0 | 6 (100) |  |
| Post arrest CPC 1 and 2, no. (%) | 1/111 (0.9) | 0/105 | 1/6 (16.7) | 0.054 |
