# Supplementary material for: Survival after traumatic out-of-hospital cardiac arrest in Vietnam: a multicenter prospective cohort study
Source: BMC Emerg Med. 2021 Nov 23;21:148. doi: 10.1186/s12873-021-00542-z (PMC8609736; doi:10.1186/s12873-021-00542-z)
Supplement: Supplementary file 4 — Additional file 4. Ethical Review Board Approval. [file 12873_2021_542_MOESM4_ESM.pdf]

Bộ Y Tế<br>**BỆNH VIỆN BẠCH MAI**<br>Số 1785/ QĐ - BM	**CỘNG HÒA XÃ HỘI CHỦ NGHĨA VIỆT NAM**<br>**Độc lập - Tự do - Hạnh phúc**

Hà Nội, ngày 30 tháng 09 năm 2015

QUYẾT ĐỊNH

Về việc: Phê duyệt đề tài KHCN cấp cơ sở năm 2015

GIÁM ĐỐC BỆNH VIỆN BẠCH MAI

- Căn cứ Quyết định số 1027/QĐ-BYT ngày 26/03/2015 của Bộ trưởng Bộ Y tế về việc ban hành Điều lệ Tổ chức và Hoạt động của Bệnh viện Bạch Mai;
- Căn cứ vào kết quả cuộc họp xét duyệt Đề tài nghiên cứu khoa học cấp Cơ sở năm 2015 của Thường trực Hội đồng Khoa học và Hội đồng Đạo đức của Bệnh viện ngày 29/09/2015;
- Theo đề nghị của phòng Nghiên cứu khoa học và Công nghệ thông tin.

QUYẾT ĐỊNH

Điều 1: Phê duyệt đề tài nghiên cứu cấp cơ sở năm 2015, tên đề tài:

Nghiên cứu khảo sát tình hình cấp cứu bệnh nhân ngừng tuần hoàn trước viện tại Khoa Cấp cứu A9 - Bệnh viện Bạch Mai

- Mã đề tài:	BM-2015-72
- Chủ nhiệm đề tài:	PGS. TS. Nguyễn Đạt Anh
- Đơn vị thực hiện đề tài:	Khoa Cấp cứu
- Thời gian thực hiện đề tài từ:	30/09/2015 - 30/09/2016
- Kinh phí thực hiện:	5000000 (vnd).

Điều 2: Phòng NCKH&CNTT và Lãnh đạo các đơn vị có đề tài được phê duyệt chịu trách nhiệm hỗ trợ, giám sát chủ nhiệm đề tài thực hiện theo đề cương nghiên cứu đã được Thường trực Hội đồng Khoa học và Hội đồng Đạo đức Bệnh viện thông qua.

Điều 3: Chủ nhiệm đề tài có trách nhiệm triển khai và nghiệm thu đề tài đúng thời hạn đã được phê duyệt. Thực hiện các quy định của Nhà nước về khoa học công nghệ và chi tiêu tài chính.

Điều 4: Các Ông/Bà Trưởng phòng: NCKH&CNTT, TCKT, Thư ký HĐKH, Lãnh đạo các Đơn vị có liên quan và các cá nhân có tên trong điều 1 chịu trách nhiệm thi hành quyết định này. Quyết định có hiệu lực từ ngày ký ban hành.

GIÁM ĐỐC BỆNH VIỆN
Chủ tịch Hội đồng Khoa học
và Hội đồng Đạo đức

BỆNH VIỆN BẠCH MAI

PGS.TS. Nguyễn Quốc Anh

Nơi nhận
- Như điều 1,4
- Lưu Phòng NCKH&CNTT

267.

BẢN DỊCH
TRANSLATION

<table>
<tr><td>

MINISTRY OF HEALTH
BACH MAI HOSPITAL
No: 1785/QĐ–BM

</td><td>

SOCIALIST REPUBLIC OF VIETNAM
Independence - Freedom - Happiness

Hanoi, 30 September 2015

</td></tr>
</table>

DECISION

On: Approval for implementing the scientific research project at the institutional level in 2015

DIRECTOR OF BACH MAI HOSPITAL

- Pursuant to Decision No. 1027/QD - BYT dated 26 March 2015 by the Minister of Health about issuing the Regulation of Organization and Operation of Bach Mai Hospital;
- Pursuant to the results of the meeting to approve the scientific research project at the institutional level in 2015 of Standing Board of the Scientific Committee and Ethics Committee in Bach Mai Hospital dated 29 September 2015;
- Based on request of the Scientific Research and Information Technology Department.

IT IS TO DECIDE

Article I: To approve the scientific research project at the institutional level, with research title: Study on management of the out of hospital cardiac arrest (OHCA) patients admitted at Emergency Department of Bach Mai Hospital

1. Research code:	BM-2015-72
2. Chaired by:	Associate Professor Nguyen Dat Anh, M.D., PhD.
3. Directly implemented by:	Emergency Department - Bach Mai Hospital
4. Duration:	30 September 2015 to 30 September 2016
5. Expenditure:	VND 5,000,000

Article II: Scientific Research and Information Technology Department and leaders of units with the approved research project take responsibilities to support and monitor the Head of research to implement the research according to the research proposal approved by the Standing Board of the Scientific Committee and Ethics Committee of the Hospital.

Article III: The Head of research will be responsible for implementing and submitting for approval of the research on schedule as approved and complying with the state regulations on technology agreements and financial expenditure.

Article IV: Heads of Scientific Research and Information Technology Department, Finance-Accounting Department, Secretary of Scientific Committee, leaders of relevant units and the person named above in Article I take responsibilities to implement this Decision. This Decision takes effect from the signing date./.

DIRECTOR OF BACH MAI HOSPITAL
CHAIRMAN OF SCIENTIFIC COMMITEE
CHAIRMAN OF ETHICS COMMITTEE
(Signed and sealed)

Assoc. Prof. MD. PhD. Nguyen Quoc Anh

Recipients:
- *As Article I, IV;*
- *Saved at Scientific Research and Information Technology Department;*

ÔNG ỨNG ĐÔ C.T.H.D T.P HÀ NỘI

LỜI CHỨNG CỦA CÔNG CHỨNG VIÊN
TESTIMONY OF NOTARY PUBLIC

Tôi **Bùi Thúy Ngọc** CMND số 164536638 cam đoan đã dịch chính xác, phù hợp với nội dung từ bản chính tiếng Việt Nam, được đính kèm theo.

*I, **Bui Thuy Ngoc,** ID card No. 164536638 hereby pledge to have translated in accuracy and consistence with the contents in attached Vietnamese original.*

NGƯỜI DỊCH
TRANSLATOR

BÙI THÚY NGỌC

Ngày: ...01.../...06.../ **2020** *(Ngày/tháng/năm)* tại Văn phòng Công chứng Đông Đô, số 101 phố Ngụy Như Kon Tum, phường Nhân Chính, quận Thanh Xuân, thành phố Hà Nội, tôi, **Nguyễn Thị Thảo** - công chứng viên của Văn phòng công chứng Đông Đô thành phố Hà Nội.

CHỨNG NHẬN

Bản dịch này do bà **Bùi Thúy Ngọc**, cộng tác viên phiên dịch Văn phòng công chứng Đông Đô, thành phố Hà Nội dịch từ tiếng **Việt Nam** sang **tiếng Anh;**

- Chữ ký trong bản dịch đúng là chữ ký của bà **Bùi Thúy Ngọc;**

- Nội dung bản dịch chính xác, không vi phạm pháp luật, không trái đạo đức xã hội;

- Bản dịch gồm **...** tờ, **...** trang, lưu một bản tại Văn phòng công chứng Đông Đô, Thành phố Hà Nội

Today, 01 / 06 / ***2020*** *(Date/month/year), at Dong Do Notary Office, No.101, Nguy Nhu-Kon Tum Street, Nhan Chinh Ward, Thanh Xuan District, Hanoi City, I, **Nguyen Thi Thao**- Notary Public of Dong Do Notary Office, Hanoi City*

CERTIFY THAT:

*This translation is translated from Vietnamese to English by **Ms. Bui Thuy Ngoc** who is a translation collaborator of Dong Do Notary Office, Hanoi City.*

*- Signature in the translation is the true and authentic signature of **Ms. Bui Thuy Ngoc**;*

- The contents of the translation are correct and do not violate the law or social morality;

- The translation includes ……sheets,….. pages, one copy is kept in Dong Do Notary Office, Hanoi City.

Số công chứng: ...07775..., Quyển số: 01 TP/CC-SCC/BD
Notarization No. :...07775..., Book No.: 01 TP/CC-SCC/BD

CÔNG CHỨNG VIÊN
NOTARY PUBLIC

S.Đ.K.H.Đ : 11 - C.T.H.D VĂN PHÒNG CÔNG CHỨNG ĐÔNG ĐÔ Q. THANH XUÂN - T.P HÀ NỘI

NGUYỄN THỊ THẢO

Bộ Y Tế
BỆNH VIỆN BẠCH MAI
Số 1785/ QĐ - BM

CỘNG HÒA XÃ HỘI CHỦ NGHĨA VIỆT NAM
Độc lập - Tự do - Hạnh phúc

Hà Nội, ngày 30 tháng 09 năm 2015

QUYẾT ĐỊNH

Về việc: Phê duyệt đề tài KHCN cấp cơ sở năm 2015

GIÁM ĐỐC BỆNH VIỆN BẠCH MAI

- Căn cứ Quyết định số 1027/QĐ-BYT ngày 26/03/2015 của Bộ trưởng Bộ Y tế về việc ban hành Điều lệ Tổ chức và Hoạt động của Bệnh viện Bạch Mai;
- Căn cứ vào kết quả cuộc họp xét duyệt Đề tài nghiên cứu khoa học cấp Cơ sở năm 2015 của Thường trực Hội đồng Khoa học và Hội đồng Đạo đức của Bệnh viện ngày 29/09/2015;
- Theo đề nghị của phòng Nghiên cứu khoa học và Công nghệ thông tin.

QUYẾT ĐỊNH

Điều 1: Phê duyệt đề tài nghiên cứu cấp cơ sở năm 2015, tên đề tài:
Nghiên cứu khảo sát tình hình cấp cứu bệnh nhân ngừng tuần hoàn trước viện tại Khoa Cấp cứu A9 - Bệnh viện Bạch Mai

- Mã đề tài: BM-2015-72
- Chủ nhiệm đề tài: PGS. TS. Nguyễn Đạt Anh
- Đơn vị thực hiện đề tài: Khoa Cấp cứu
- Thời gian thực hiện đề tài từ: 30/09/2015 - 30/09/2016
- Kinh phí thực hiện: 5000000 (vnđ).

Điều 2: Phòng NCKH&CNTT và Lãnh đạo các đơn vị có đề tài được phê duyệt chịu trách nhiệm hỗ trợ, giám sát chủ nhiệm đề tài thực hiện theo đề cương nghiên cứu đã được Thường trực Hội đồng Khoa học và Hội đồng Đạo đức Bệnh viện thông qua.

Điều 3: Chủ nhiệm đề tài có trách nhiệm triển khai và nghiệm thu đề tài đúng thời hạn đã được phê duyệt. Thực hiện các quy định của Nhà nước về khoa học công nghệ và chi tiêu tài chính.

Điều 4: Các Ông/Bà Trưởng phòng: NCKH&CNTT, TCKT, Thư ký HĐKH, Lãnh đạo các Đơn vị có liên quan và các cá nhân có tên trong điều 1 chịu trách nhiệm thi hành quyết định này. Quyết định có hiệu lực từ ngày ký ban hành.

GIÁM ĐỐC BỆNH VIỆN
Chủ tịch Hội đồng Khoa học
và Hội đồng Đạo đức

PGS.TS. Nguyễn Quốc Anh

Nơi nhận
- Như điều 1,4
- Lưu Phòng NCKH&CNTT
